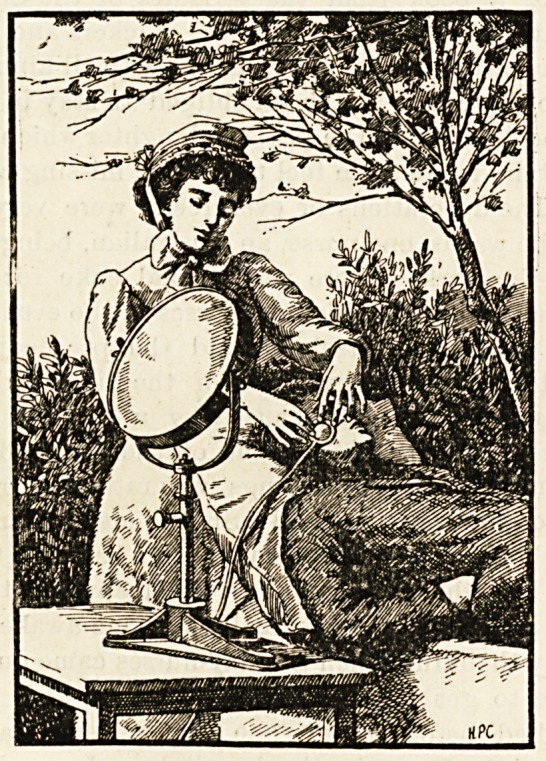# The Hospital. Nursing Section

**Published:** 1903-04-25

**Authors:** 


### The Hospital.

### Nursing Section.

Contributions for this Section of "The Hospital" should be addressed to the Editor, "The Hospital" Nursing Section, 28 & 29 Southampton Street, Strand, London, W.C.

No. 865.—Vol. XXXIV. *SATURDAY, APRIL* 25, 1903.

### Notes on News from the Nursing World.

### THE REPORT OF THE DEPARTMENTAL COMMITTEE.

It is understood that the paper which Dr. Downes is to read at the West Midland Conference at Malvern on the 12th of next month on "Nursing the Sick Poor in Workhouses" will in effect be a defence of the Report of Departmental Committee, of which he was the most important member. Dr. Downes, we hope, will be able to make the welcome announcement that in deference to the overwhelming preponderance of opinion in the nursing world, the proposal of the Committee to create an order of "qualified nurses" of one year's training will be abandoned. With all his ability and experience he cannot make out a case for this recommendation.

### DEATH OF MRS. HECKFORD.

We regret to announce the death, at Pretoria, of Mrs. Sarah Heckford, whose contributions during the war of Boer treatment of the natives and the condition of the Concentration Camps excited so much attention. But for our readers the notable feature in the career of the deceased lady is the fact that in the cholera epidemic of 1866, Miss Sarah Goff, with her sister, who is still alive, volunteered to act as nurses in East London. It was in the Cholera Hospital at Wapping that Miss Goff met Dr. Nathaniel Heckford, who had volunteered from the London Hospital for the same cause, and whom she afterwards married. The marriage took place when the epidemic was over, and it was then that together they originated the admirable institution in Shadwell, which is now known as the East London Hospital for Children. Dr. and Mrs. Heckford secured the possession of an empty warehouse at Ratcliff Cross, and having furnished it with 10 little beds, and inscribed over the doorway the title "East London Hospital for Children and Dispensary for Women," they took up their own residence there in rooms on the first floor. There, too, they lived and laboured until their happy wedded life was suddenly terminated by Dr. Heckford's premature death. He never saw the present hospital, and his young widow, overwhelmed by her bereavement, left the work in the hands of a capable committee, and went to live in Naples. Her after days were spent in Italy, India, and the Transvaal, and were full of interest and adventure. It will never be forgotten that in the time of her youth, when she had the command of wealth, and might have lived in luxurious ease, she voluntarily devoted herself to a ministry of love among the toiling masses in the East End of London.

### A NEW DEPARTURE FOR THE COLONIAL NURSING ASSOCIATION.

It is a compliment to the Colonial Nursing Association that the committee have lately been asked by the Foreign Office to select two nurses for service in East Africa. At the outset they will probably be stationed at Mombasa, the terminus of the new Uganda railway, but no doubt in time, as the European population extends, nurses will be sent inland up the line as far as Port Florence, on the Victoria Nyanza, the western terminus of the railway. At the instance of the committee, who were asked to make a selection, Miss E. L. Jardine has lately been appointed matron of the Home for Freed Slaves in Northern Nigeria. The home was founded by Sir Frederick Lugard to meet a serious difficulty in connection with the children who are rescued from the hands of the slave dealers, and it will shortly be moved to Zungeru, his present headquarters.

### NIGHT NURSES AT THE NATIONAL HOSPITAL.

Hitherto the night nurses' quarters at the National Hospital for the Paralysed and Epileptic in Queen Square, Bloomsbury, have been on the top floor of the wing where the wards of the male patients are situated. But when the home for them is opened in the beginning of May the night nurses will not only have much more comfortable quarters, but their former bedrooms also will be available for fresh isolation wards. The new home, in which there will be accommodation for 13 nurses and a reliable housekeeper to look after them, is a private house in Powis Place, which has been admirably adapted for the purpose. It can be reached from the hospital through the out-patients' department and also from the wards, where the architect has cleverly cut a passage through the centre of the roof of a ward, and thus connected the two buildings on the top as well as on the bottom story. The night sister has a cosy bed-sitting room; most of the nurses have separate rooms, but there are of necessity a few bedrooms for two nurses, because it was essential to adapt the house with as few alterations as possible. For these special furniture has been designed and each nurse has her own chest of drawers and washing-stand, bed and locker, with a dual wardrobe, each side quite separate. The rooms are all painted in pale green, with green linoleum on the floor and bedside rugs, and the electric light has been installed throughout the premises. The kitchen is fitted with a new range, the bathroom is evolved from the original back kitchen, with hot and cold water laid on from the hospital. New drains have been put in all over the house, and the floor has been freshly cemented. There is a pleasant sitting-room on the ground floor painted and upholstered in terra-cotta. The housekeeper will be always at hand to get a cup of tea for a nurse off early for a holiday or one breakfasting in bed; but to prevent nurses using spirit stoves in their bedrooms a special gas-stove, slate-surrounded so as to avoid all risk, has by request of the lady superintendent been placed on one of the landings, with tea-things kept in a cupboard underneath. The addition of a home entirely devoted to the night nurses quite away from the hospital or from day nurses' quarters, has obvious advantages.

### A MATRON WHO COULD NOT KEEP BOOKS.

The Guildford, Godalming, and Woking Joint Isolation Hospital Board, have been compelled to make a remarkable confession. It is to the effect that the matron of Woodbridge Hospital, whose services they have lately dispensed with, was appointed without the Board knowing anything about her abilities. They were not even aware whether she could keep the books. The result was that she had to seek the assistance of a District Council office clerk, who used to go to the hospital in the evening and have tea with the nurses. The clerk has since sent her in a bill for £10 for his services, which she refuses to pay. This is why the matter was brought before the Board and their laxity in not taking the trouble to ascertain the qualifications of the lady they selected for an important official post is deplorable. We agree with the member who urged that whatever there is to pay, it should, in the circumstances, be paid by the Board.

### TRAINING NURSES BY CORRESPONDENCE.

The matron of a sanatorium in California, United States, writes to us to express the hope that "an evil which is rapidly developing in America will not spread to the old country." She refers to the system of training nurses by correspondence, and she encloses an advertisment from a Detroit paper, in which the Detroit School of Nursing intimates that, "a thorough practical course of instruction and training is given by an institution established under the laws of the State of Michigan, which you can take at home by mail at small expense." It is added that "diplomas are issued," even, we suppose, though the "nurse" has not seen so much as the door knob of a hospital. Moreover, we learn that owners of these "diplomas" command the same salary as the nurses who have patiently gone through their training. The most serious feature is, of course, that institutions issuing such certificates are sheltered by the State. We do not think, however, that the old country is in any danger of being invaded by an order of nurses trained by correspondence. In that respect, at least, we are likely to remain behind America.

### IMPROVEMENTS AT CHORLTON.

The foundation-stones of two hospital pavilions and of an annexe to the Nurses' Home at Withington have just been laid. When the latter building is finished, the accommodation for the nurses in the employ of the Chorlton Board of Guardians will be doubled. The existing building, erected in 1885, has provision for 60 nurses. It is due to the Chorlton Guardians to say that they are in the van of progress so far as nursing in workhouse infirmaries is concerned. Mr. Jenner Fust's return for last year relating to the sick in workhouses shows that on January 1st last there were in the Chorlton Union Hospital 771 acute sick cases. These were attended by 50 day nurses, and at night by 24 nurses, an average of about 1 in 15 during the day, and 1 in 32 during the night. Mr. Ramsden, who laid one of the foundation-stones of the new extensions, said that the term workhouse was a misnomer, and that the institution was really a hospital for physical and mental diseases, and a refuge for the aged poor. It is a pity that this admirable view is not taken and acted upon more by guardians generally.

### HOW NOT TO MANAGE A NURSING ASSOCIATION.

It is not in the least surprising that the accounts of the Watford Nurses' Fund show a balance of £44 on the wrong side. At the annual meeting of subscribers Mrs. Panton pointed out "the unbusiness-like way" in which the association is managed. As the accounts of the Watford fund have neither been audited nor signed, and as there is not even a list of subscribers a much more emphatic adjective might fitly have been used. No organisation that does not have its accounts audited and signed can expect to gain the confidence of the public, and in the absence of a published list of subscribers it is perhaps astonishing that the receipts of the Watford Fund were as much as £185 last year.

### HORSEPLAY IN PRIVATE NURSING.

It is very essential that private nurses should not be without recreation. But the kind of recreation which was provided for Miss Cundall in the house at Wood Green, where she was nursing the mother-in-law of the tenant, cannot be commended. The story of the nurse, as told in Tottenham Police Court in support of a charge of assault, is that on her return to her duties on the evening of Sunday, March 29, she was admitted by the master of the house, who dragged her into the dining-room, lifted her up like a child, and dropped her so heavily on the floor that she subsequently had to undergo an operation. According to her further statement, when she retired to rest she found the sleeves of her nightdress stitched together, her hat-pins broken, and the candle in the bed. Then the door of her room was opened and soda-water having been squirted over her, a wet sponge was thrown at her. The bed-clothes were also pulled off her by a string which communicated with the bedroom of the defendant and his wife. All this was admitted, but the defendant and his wife asserted that they were only joking, that Miss Cundall was not injured, and that she said she enjoyed the joke immensely. As the magistrates, after five hours' hearing, dismissed the summons, the conclusion is that they were satisfied that the nurse was a party to the horseplay of which she complained. If she resented the treatment to which she was subjected, she should have left the house the next morning, instead of remaining for a couple of days.

### NURSING IN A FLOATING HOSPITAL.

The Boston Floating Hospital has been in existence more than eight years. Started in a barge with the idea of getting a number of sick babies and their mothers out in the fresh air for a day, with one nurse and an assistant to direct the mother in the care of the little ones, it has steadily developed into an admirably equipped hospital. There is now a considerable medical staff, nurses are trained, and in 1902 sixteen received certificates. The excursion element has been almost eliminated, and between fifty and sixty of sick Boston babies, sent by the physicians of the city, are carefully nursed in the fever wards, which are full of cribs and cots. In 1902 no fewer than 1,902 were admitted between July 8th and September 15th, and as many more were rejected for lack of room. The system of nursing is much the same as in ordinary American hospitals. The hours of duty are from 7 A.M. to 8 P.M., with definite times for meals and rest, and regular hours off duty on certain days. Meals are served in the little dining-room, and for rest a portion of the upper deck is reserved and furnished. The nurses on day duty sleep in rooms provided for them in East Boston, within easy walking distance of the wharf where the floating hospital ties up at night. While the instruction given to nurses is not novel, there are peculiarities in its application. For example, all diarrhœal cases are considered contagious, and the nurses who come in contact with them wear elbow-sleeves and frequently scrub hands and arms with a solution of cyanide of mercury. All flies are screened away, and all diapers as soon as possible destroyed. Extra precautions are observed to prevent the contamination of food, and physical examinations of each child are made in which the nurses assist and are permitted to ask questions. If there are post-mortem examinations one or two nurses are allowed to be present, and thus they are afforded a chance of learning why their efforts have failed as well as of becoming familiar with the lesions of disease.

### WORKING MEN AND SICK NURSING.

It is a reflection on the working men of the second city in the Empire that last year they only contributed £56 16s. to the funds of the Glasgow Sick Poor Nursing Association. They can scarcely plead that they do not know of the existence of the organisation, but it is possible that they have overlooked its wants. It was founded 27 years ago, and it has been an immense benefit to thousands of families. In 1902 its expenditure was £3,608, but its ordinary income was only £2,815. This means a deficit of £793. It may be urged that the working men cannot be expected to convert the debit into a credit balance, but it is suggested by one of their number that such an achievement would not be in the least a strain upon their resources. He pleads that even if they gave 3d. each a year the present deficit would be covered, while 6d. each would provide a handsome surplus and enable the directors of the Association to add to the number of nurses in the districts where more are specially needed. It is proposed as means to these ends that a committee of working men should be formed, and that they should undertake to secure an annual collection from all the public works and warehouses in each district. The proposal seems to be exactly what is required to meet the necessities of the case, and the result will be watched with interest on this as well as on the other side of the Tweed. Experience teaches that if working men are systematically invited to maintain the nurses who minister to their own people, they generally respond in a satisfactory manner; though, like other persons, they often fail to render help unless they are individually asked to do so.

### DERBYSHIRE HOSPITAL FOR SICK CHILDREN.

A double loss is about to be sustained by the Derbyshire Hospital for Sick Children. The honorary lady superintendent, Miss Cupiss, and the head nurse, Miss Clarke, will retire in June. Miss Cupiss, whose retirement is due to ill-health, is one of the founders of the hospital, and has been one of its most devoted friends. The work was commenced in a house in Derby in the year 1877 with seven beds, and the present excellently-equipped hospital owes its success in no small degree to the untiring and unselfish labour of love of the lady superintendent, who, in addition, had contributed handsomely to the funds. Many little sufferers have to thank Miss Cupiss for her generosity in providing at her own expense surgical appliances and other necessities. The working men's committee, who hold her in great respect, have passed a resolution expressing their deep regret at her resignation and their appreciation of her work. Miss Clarke, the head nurse, has filled her post for nearly 25 years, and her devotion and tact have gained her the confidence and esteem of every one connected with the hospital. She has been an unfailing friend to the inmates of the institution.

### SALISBURY NURSES' HOME.

In eulogising the work of the Salisbury Nurses' Home at the annual meeting, the Dean of Salisbury said that from his own connections and relations he knew what a boon it had been to have a paying hospital in Fitzroy Square, and the special private nursing room at St. Thomas's Hospital. The report of the institution shows a deficit of £16, which is accounted for by the reduced charges at which some of the patients were nursed. This, we are afraid, is an almost inevitable result of the system. As the hon. secretary observed, the Home has two objects in view, one of which is to prescribe skilled nursing for the public both indoors and out of doors, while the other is to nurse the sick poor, the cost of the latter being covered, as a rule, by public subscriptions. But there are evidently exceptions to the rule, as in 1902, and we suspect that the cause is to be found in the mistaken impression that the fees of the paying patients cover the expenses of the undertaking.

### THE ROYAL BRITISH NURSES' SETTLEMENT.

A very successful sale was held by the kindness of Miss Scott in aid of the funds of the Royal British Nurses' Settlement at Inglewood, Bournemouth, on Monday. The stalls were furnished with articles remaining unsold from the sale held at Lord Brassey's house last year, and a complete clearance was effected. A provision stall, with dairy produce, cakes, jam and other good things, was a very popular feature. The total proceeds amounted to nearly £58, which have been handed over to the honorary treasurer.

### The Nursing Outlook.

" From magnanimity, all fear above;
From nobler recompense, above applause,
Which owes to man's short outlook all its charm."

### THE NURSE IN PRISON.

Not as prisoner, but as warder. It probably doesn't sound attractive at first, but possibly on further consideration it may show a new outlet for the trained and devoted woman. For gradually we are coming to recognise that disease of the soul (commonly called sin) is akin to disease of the body; that the two often go together, and that the prisoner is often more feeble than faulty, more the victim of herself than the victimiser of others. This has been practically acknowledged by the appointment of Dr. Donkin, formerly of the Shadwell Children's Hospital, as one of the Prison Commissioners, and more especially in the late arrangements at Holloway as the great prison for women. Until last autumn the women under short sentence and under remand alone went to Holloway, and the others were sent to Wormwood Scrubs; but now Holloway is reserved exclusively for women, and the governor and the assistant-governor are both medical men. So far nothing very great has been achieved under the new *régime*, but time is wanted for all improvements, and the right workers are wanted in all fields. We need some Florence Nightingale to go and train as a warder, and make the tendance on the sick of soul as holy as the tendance on the sick of body. For it is the warder who comes closest into contact with the prisoner; the governor, and the chaplain, and the lady visitor are all fulfilling their duty no doubt, but the constant daily watching, and example, and keeping of a high ideal must ever remain with the warders. And example is the chief means of educating the sick of soul; to have constantly before one an example of uprightness and punctuality, and courtesy, and self-control, probably teaches a prisoner far more than any sermon can do. Then is it not worth while for women who have sympathy with the most unfortunate of their sex, to devote their lives to the bettering of prison conditions in England? If it is well to be a hospital nurse and minister to the diseased of body, is it not better to be an asylum nurse and minister to the mind diseased, but best to be a prison warder and minister to the sick of soul? But it cannot be done without sympathy, without love and patience. It needs a knowledge of the trials of these women's lives, and feeling for their lack of opportunity, an appreciation of their helplessness and hopelessness. In one year alone 400 women were remanded to Holloway on the charge of attempted suicide. Does not the misery indicated by such a fact appeal to those who are too ready to pass by on the other side when they see thieves and sinners? If ever there was an opening for woman's work for woman it is to be found now in the prison world, and any nurse applying to Dr. Donkin, at the Prison Commission at the Home Office in Whitehall, may be sure of a courteous reply; for he has at heart the improvement of the condition of the warders' life and the elevation of their status in general. Of course the work must be taken up at the beginning; but the actual training is only for three months as against the three years' hospital training, and there is at present no rush of well-educated women into the ranks of warders. The pay is small at the commencement, but there are a few good posts to be won, and there is the glory of being a pioneer. The hours are not long, and the holidays are regular; the housing of the warders at Holloway leaves much to be desired at present, but we can trust Sir Evelyn Ruggles-Brise—the Chairman of the Prison Commission—to see to that in time.

Now a word as to the special qualifications and knowledge necessary to the nurse who would help the sick in soul. She must begin by recognising that the great aim of prison work is to reform and not to punish; to make the prisoners more hopeful and useful, and not to make them more miserable and mechanical. It is here that our prisons fall so far behind those of the United States and other countries. In our hard, cold English way, we have always held it just to punish the prisoner, quite forgetting the difference between the sinner and the criminal. For a boy who rides his bicycle on the path, a woman who fails to send her child to school, may become prisoners, because they have broken the law of the country, not because they have broken the law of God and become sinners. And then we have been such terrible Pharisees that once a person has been to prison we have condemned them to distrust for all time, and made them a thousand times worse than they need be. Surely no public sin has been greater than this, that we have so run our prisons, that once the contamination of them has fallen on a man or woman, that man or woman has been hopelessly ruined. Once a prisoner, always a prisoner, or always an in-and-out. What bitter mockery to imagine we are Christians who would save the sinner, when, on the contrary, we do our best to make him worse. What good can it do a woman who has attempted suicide, or a man who has used obscene language, to be shut up in an ugly dim cell, with their own ugly dim thoughts? In America they would be put into a big building flooded with light and air; they would be cheered by companionship, and taught a trade, and fed scientifically to restore their bodily losses. And when they left a situation would be found for them, and they would set forth better and brighter and more fitted to face the world that had bested them for a time. Oh that in our English prisons some American and saving methods might be adopted, and that the 800 women prisoners now in Holloway might be ministered to by those who would try to save them, and not to punish them!

### Lectures on Medical and Surgical Nursing.

By John Hopkins, F.R.C.S., Medical Superintendent of the Central London Sick Asylum District Asylums, Cleveland Street, W., and Hendon, N.W.

### LECTURE VI.—TUBERCLE AND SYPHILIS.

*Tuberculosis* is caused by a growth in the tissues of a bacillus, which is capable of surviving the onslaught of the leucocytes in certain individuals. It is always very local to begin with, and generally spreads from one part to another very slowly. When it has succeeded in getting a foot-hold in the body, it may, even after it has done great damage, be overcome and destroyed by the activity of the white blood cells. When it affects the glands of the neck it gives rise to scrofula, an older name for which is King's Evil, as it was believed that a touch from a King's hand would effect a cure. It is a disease that may be prolonged over many years. The most effectual remedy is to excise the glands affected. When left to nature, it often gets well, but leaves unsightly scars behind. The lungs are a common seat of the growth of this bacillus. Wherever they are, they give rise to the growth of a little mass of tissue round them, called a grey granulation, as it is the size of a small grain of seed and has a grey translucent appearance post-mortem. Many of these granulations growing together may form so dense a mass as to cut off all blood supply, and then the centre of this mass degenerates and forms a yellow bloodless material which softens and may be coughed up leaving a cavity, the walls of which are surrounded by tubercular growth, which ulcerates and comes away, causing an enlargement of the cavity, and exposing blood vessels, which sometimes burst and give rise to hæmoptysis or blood spitting.

When tubercle grows in the lungs it often leads to the subject's death, but there is no reason why a cure should not be effected, especially in early cases. Growing in the bones, the bacillus causes ulceration of the bone, which may or may not be accompanied by suppuration. The tubercle bacillus does not necessarily cause the formation of pus, and the suppuration seen so often in tubercular disease is generally due to the presence of other microbes besides. When the tubercular ulceration takes place in the bodies of the vertebræ, these melt away, and allow the upper part of the body to fall forwards. This produces an angular curvature of the spine. Abscesses formed at the site of this caries, as the ulceration is called, are generally cold, and they may burrow a long way along the spine or in other directions before they point on the surface. When these abscesses are opened the temperature of the patient may rapidly go up. This is due to the introduction of new kinds of microbes. The abscess is then said to be infected septically, and the larger the abscess the greater is the danger to the life of the patient. If the abscess be opened antiseptically and great care be taken afterwards in the dressing of the wound not to let other bacteria in, this dangerous inflammation in the abscess will not occur. The awful responsibility of dressing such a case with due precaution cannot be too much impressed upon you.

Young children, especially when infected by tubercle, are likely to have the bacillus conveyed by the blood to the meninges of the brain, where, after growing for some time and giving rise to numerous grey granulations, it excites a fatal tubercular meningitis. The bacillus, when the skin is inoculated with it, causes the growth of a spreading wart, known as anatomical wart, because it is most frequently found in those who have the handling of tuberculous organs in the post-mortem room. Lupus is another skin disease due to the tubercle bacillus. In the nursing of cases of tubercular disease, the general principles that must guide you are those which serve for the improvement of the general health. Fresh air night and day in a locality where it is purest, and most likely to have present in it that condensed state of oxygen which is known as ozone, the patient spending all the twenty-four hours of the day in the open-air, is now recognised as a poweful remedial agent. At the same time abundance of good plain food, that is easily digested, is required, as the patient acquires a good appetite which should be satisfied. Warm clothing sufficient to prevent the patient getting cold, a light and cheerful occupation, pleasant and happy surroundings, are all necessary to put the patient into the best condition to combat the disease. The white blood cells become better nourished, more active and capable of enclosing and destroying the numerous bacilli that invade the patient's tissues.

*Syphilis* resembles tubercle inasmuch as when its microbe invades the tissues it lives and grows there perhaps for a prolonged period. We have seen that in the specific fevers the active living micro-organism disappears entirely in a few weeks, destroyed by the antitoxins of the blood. There is good reason to believe that the same thing takes place to some extent in tubercle and syphilis, the micro-organisms that are the cause of them being destroyed in certain individuals. But this is exceptional. As a rule the blood-cells are incapable of doing this in tubercle and syphilis.

There is an incubation period in syphilis of three to five weeks from the time of inoculation. Then the rash appears, and the lymphatic glands are enlarged and tender wherever they can be felt. The skin in this disease may exhibit almost every kind of known rash. At a later period, perhaps after years, other symptoms may appear; growth of a form of tissue possessed of low vitality and forming lumps occurs. These are called gummata. Lumps of the bones called periosteal nodes are often very painful. A destructive ulceration of the nose or palate is common. The brain, cord, and membranes and their blood-vessels may be invaded, and a growth of inflammatory tissue takes place causing compression of various parts, pain and paralysis. There is no part of the body that may not suffer in one way or another.

Fortunately there are drugs capable of destroying the syphilitic micro-organisms. They take a long time to do this though, and patient perseverance in the administration of the medicine prescribed or in using the outward applications ordered is necessary. Patients are apt to become impatient under the circumstances and too often cease to take the medicine before they should do.

Syphilis is peculiar in that it is transmitted from parent to offspring, and may after birth show its presence, not only in modifying the growth of the child, but in various symptoms of active disease. Rashes and a free secretion of mucus in the nares causing snuffles are the first signs to appear. At puberty the cornea of both eyes may become acutely inflamed.

In dealing with this congenital form of syphilis, mercurial inunction is often adopted. A small quantity of blue ointment is rubbed into the soles of the feet, or into the knee, or into the waist where it can be covered in by socks, a bandage, or a binder. Infants troubled with snuffles cannot continue to suck for any length of time, for being unable to breathe properly through the nose, they have to drop the teat from the mouth to draw breath. When a feeding-bottle is used, the nurse has to constantly replace the teat in the child's mouth, so that it may get a good meal. This condition makes infants fretful when feeding. Congential syphilis needs the same persevering treatment that has already been mentioned under the acquired form.

### The Nurses of the Japan Red Cross Society.

BY THE MANAGERS.

The Japan Red Cross Society has the power to summon its reserve nurses in sufficient numbers when the society has to undertake relief work in time of war, or on the occasion of a public disaster. In ordinary times they are allowed to live in their respective homes, nursing as they choose. But some of them get themselves appointed as nurses in ordinary to the hospital of the society in order that they may further prosecute their studies, while not a few others enter the Nurses' Exterior Service Department, which is a special establishment for according those nurses means of support as well as opportunities for improving their technical knowledge.

The nurses of the society do not make it their object to earn their living by the practice of their profession. They desire to satisfy their patriotic inclinations by partaking of the general work of the society in time of war so far as it concerns the succour given to military patients. Therefore, whenever we are disposed to start our relief work, they vie with one another to report themselves in response to our summons. So did they rally to our satisfaction both in the case of the Japan-China War of 1894–1895, and in the case of the North China troubles of 1900. This is a fact which we desire to make known to the general public of Great Britain through the agency of The Hospital.

The number of the reserve nurses trained by the headquarters and the branch establishment of the society is 1,518, of whom 195 have been educated at the headquarters, while the number now under training as student nurses is 659, of whom 120 belong to the headquarters.

### Nurses at the Hospital.

The number of sick-rooms in the hospital is 58, with 199 bedsteads, while the nurses on duty in these wards number 200, including the student nurses. The rooms are divided into six sections, to each of which one or two matrons and not less than six nurses are attached. The matrons are selected from among the senior, more proficient, and better conducted nurses of the hospital. The principal matters which a matron is required to take charge of are as follow:—

1. To inspect the services of nurses under her direction to transmit the orders of her superiors to the members of the section; and to communicate all that transpires in her section to the superiors.

2. To acquaint the members of her section with the directions and instructions of the doctor-in-charge with respect to nursing and to see them carried out.

The Japanese Red Cross Society's Hospital.

3. To examine and keep the sick-room journals and similar documents.

4. To see the bandage material, surgical instruments, bedclothes of the patients and ward furniture well cared for, and to distribute medicines and foods to the patients.

5. To pay due attention to the rooms being kept clean, and to see the electric light apparatus and stoves well taken care of.

The nurses attached to the wards have to faithfully discharge their duties in accordance with the instructions of their superiors and are also required to hold themselves duty bound to guide the student nurses within the sphere of their duties, showing them practically examples to be followed.

The matron and nurses of each section have to serve from 8 A.M. to 8 P.M. each day. Besides, the nurses, in varying numbers from two to five per room, in accordance with the number of the patients, have to take night duty by turns,

THE NURSES OF THE JAPAN RED CROSS SOCIETY—*Continued.*

one set from 6 P.M. to 1 A.M. and the other from 1 A.M. to 8 A.M.

The nurses are all required to reside in the dormitories attached to the hospital and not to leave the hospital grounds without sufficient reason except on Sundays, national holidays, and certain anniversaries. In the dormitories facilities are afforded to the inmates for receiving lessons in house-keeping and foreign languages, principally English. For the keeping of good discipline and for the purpose of control over the sanitary matters of the dormitories, a superintendent nurse is appointed, while the

A Japanese Red Cross Nurse.

matrons are required to assist the superintendent in the respective dormitories to which they are attached.

The nurses on duty in the hospital wear uniforms established by the Society. They have to observe the rules of decorum and to maintain proper discipline and reputable character.

The allowances to the nurses connected with the hospital vary from 12 to 25 yen per month. The uniform and working costumes are given out by the Society.

Nurses in the North China Disturbances.

Out of the nurses despatched by this society during the North China disturbances of 1900 to minister to the sick and wounded soldiers, those who served in the military hospitals at home were one superintendent nurse, 16 matrons, and 163 nurses. The French and Austrian soldiers, wounded or sick, who were entrusted to the Military Hospital at Miroshima for treatment, were all tended by the nurses of the society. As to the service at sea, a superintendent nurse and 10 nurses, besides a number of male nurses, were placed on board the *Hakuai-Maru* and *Kosai-Maru*, which first sailed in July 1900. They were despatched for the purpose of utilising their services in connection with the more serious cases. These nurses had the care of the sick and wounded who were transported in those ships from Taku, China, to Njina in Miroshima ken. The patients thus transported were 1,498 Japanese and 98 French soldiers in the case of the *Hakuai-Maru*, and 1,292 Japanese, 25 French, and two Austrian soldiers in the case of the *Kosai-Maru*. Each of the two ships made seven voyages, and both were released from duty in the latter part of November, when the mouth of the Teiho commenced to freeze. It is a source of great satisfaction to us to be able to say that these nurses acquitted themselves splendidly in the pursuance of their onerous duties from the time of great heat in July till November, when the harbour of Taku became ice-bound, and it is a pleasure to add that not one of them fell ill during that period.

### Four Months' Training in a Maternity Hospital.

BY A NURSE IN THE NORTH.

Being desirous of obtaining a certificate of midwifery from a good training school, I last summer entered my name at a hospital in a northern town, and some two months later, when a vacancy occurred, repaired thither for a course of four months' training. I arrived late at night, after travelling for twelve hours, to find that my luggage had been left behind at one of the stations where I had changed; however, after being reassured by the officials that as it was correctly addressed it would be forwarded to me, I got a cab and set off for my destination. The cab-driver was not very sure of the locality, and after driving for what seemed to me a long time, he stopped, and coming to the window announced, "I can no tak the machine down the brae, but yon's the place," so on foot I proceeded to the hospital door.

A Chilling Reception.

My reception was rather chilling. A night-nurse opened the door and seemed most surprised to see me. She requested me to follow her upstairs, which I did, and up and up we went, the quietness of the night broken by the wailing of infants. There was a bright light in a room on the second flat, and the sound of voices proclaimed even to a newcomer that a case was going on. At last we reached the third flat, and my guide, who had not been very long in the hospital herself, proceeded to find my bedroom. Three doors were opened before the right one was discovered, and then a new difficulty presented itself: by an oversight no sheets had been left out for me; after a search one was found, and with that I made up my bed. The night-nurse had left, but returned presently with the welcome intelligence that she had prepared some supper. I had undressed in the interval, and in a borrowed dressing-gown I adjourned to the nurses' kitchen and partook of my first meal in Scotland. Next morning, as I had no uniform with me, I was allowed to remain in bed until breakfast-time. After that meal I went to the station, and finding my boxes had arrived safely, I got a cab and returned with them in triumph. No boxes were allowed to be taken upstairs, so I had to unpack in the hall and carry my clothes up 85 stairs, and to add to my discomfiture there were four or five students standing about waiting for a class.

### The First Lesson in Sponging.

I had just finished putting everything away when a nurse came to see if I was ready to come to the wards to receive my first lesson in "sponging." There were four other new nurses, and we were all shown the elaborate precautions undertaken to prevent any risk of sepsis; at first it was very novel to us, but long before our training had expired use had become second nature, and we did not forget any of the details by which our hands were sterilised. Then we were taken to the linen-cupboard and shown where the different articles were kept, and after that I was appropriated by one of the nurses for her ward, where I remained for some ten days. Having had some previous training, I did not find my first appearance in the "labour ward" the serious ordeal that a girl entirely new to hospital life does, although, being in close proximity to the anæsthetist, I felt rather sick. It was one of the rules of the hospital, and it seemed to me a very good one, that in a case of normal labour all the new nurses were sent into the labour ward to watch proceedings.

### A Day Special.

At the expiration of a week two of the nurses who came the same day as I did were put on night duty, but it was not for two days more that I was moved from my first ward, and then it was to be "day special" with a case of Cæsarean section. The woman had come in the night before, and at 3.30 A.M. most of the nurses in the hospital were roused to get things ready for immediate operation, as labour pains had commenced. My duty was to prepare hot-water bottles, and not being thoroughly acquainted with the various wards it was rather a hunt to find them; however, in a very short time all was ready, but after the doctor who was to operate had arrived he decided to postpone it for a few hours. The operation took place after breakfast, and my share of the work was to see that the staff-nurse had plenty of hot water for the lotions, but in spite of being so occupied I saw the operation splendidly. As soon as all was over and the room cleared I was installed in charge of the patient, and continued to look after her and the baby for four days, when she was moved into an adjoining ward with another case of Cæsarean section, and one nurse attended to the two patients. The next morning I began work as one of many in a big ward, but owing to several new cases coming in, it was decided to open another ward in the upper flat, and after washing a new baby, I was sent to take charge of it. I had only two patients and a baby to look after, but by the next day the number had increased to three patients and three babies, and as nurses were scarce I was single-handed. This did not last, and next day the ward was in charge of one of my seniors, and I was moved and put on special night duty.

### An Anxious Period.

I had charge of the two cases of Cæsarean section I have previously mentioned. Only one of the babies was living, but he was a little darling, and my special pet during the time he remained in the hospital. I seemed to be going on splendidly when my luck turned, and owing to a boil I had to be removed from actual contact with the patients, and for some nights my duties were to prepare gruel for the patients, bath new patients, answer the door, and cook the nurses' meals. As I did not get better I went away from the hospital for a short time for change of air, and very much I begrudged the time wasted; but "it's an ill wind that blows nobody good," and I took advantage of the leisure to study my text-book and get a knowledge of the theory. On my return to the hospital I started to "get up my cases," and owing to several abnormal cases which required the intervention of the house-doctor, I was some ten days before I obtained the three deliveries which a nurse usually got before proceeding to work on the district. I must confess that at this period of training I felt terribly nervous, a feeling which is, I believe, very general. When not in the labour ward occupied with a case, I was looking after two patients in a small ward, and I greatly valued being able to attend to them during the whole of the time they were in the hospital, and to watch their progress. Both were cases requiring douches, which meant additional experience. I became very attached to one of them, and am sorry that I was not able to hear of her again after her departure home.

### A Festive Interval.

These experiences occurred about Christmas time, and in passing I will devote a few words to a description of how we spent the festive season. The matron very kindly took three of the nurses, who were communicants, to an early Celebration with her, which helped us to realise that it was really Christmas, although in Scotland the festival is not observed as it is this side of the Border. At dinner-time, when the fare was roast beef and plum-pudding, the tables were very nicely decorated, and beside every nurse's place was the present of a book from the "Ladies of the Committee." Great amusement was caused by the crackers and the subsequent decoration of the nurses' heads with all kinds of head-gear. To those who had been obliged to stay in the wards during the first dinner the peals of laughter which proceeded from upstairs made them feel they were missing a good deal of fun. The decorations of evergreens were very pretty in some of the wards, one nurse, an Australian, being especially clever at the work. There was special cake for tea and a dance in an empty ward for the nurses in the evening. This was just amongst ourselves, and the proceedings were redeemed from dulness by some of the high-spirited ones; special amusement being caused by a cleverly humorous imitation of Paderewski by one of the Australian nurses. My enjoyment of the proceedings was rather marred by the fact that on my way up to bed "Sister" met me and told me that she should want me to sit up with a bad case which had just been brought in. I was terribly sleepy, but the serious condition of the patient effectually kept me awake, although I was very thankful when the day-nurses came on duty and I was free to get my breakfast and go to bed. The poor woman died before night, one of the few deaths which occurred while I was in the hospital, so I was allowed to sleep on undisturbed.

*(To be continued.)*

### To Nurses.

**We invite contributions from any of our readers, and shall be glad to pay for "Notes on News from the Nursing World," or for articles describing nursing experiences, or dealing with any nursing question from an original point of view. The minimum payment for contributions is 5s., but we welcome interesting contributions of a column, or a page, in length. It may be added that notices of appointments, entertainments, presentations, and deaths are not paid for, but that we are always glad to receive them. All rejected manuscripts are returned in due course, and all payments for manuscripts used are made as early as possible after the beginning of each quarter.**

### The Finsen Light Treatment.

### I.—INTRODUCTORY.

If you pass a ray of light through a glass prism it comes out broken up into a series of colours similar to those of the rainbow. If a nurse has never seen this done in a spectroscope, perhaps if she recalls the cut-glass chandeliers of her childhood and the gorgeous colouring they gave, she will understand what is meant. The order of the colours is thus:—

Visible spectrum. { Red. Orange. Yellow. Green. Blue. Indigo. Violet. }

Indigo., Violet. and Ultra violet } Finsen rays.

Invisible . . Ultra violet

Now, away back in 1839-40, when photography was in its infancy, men's minds were much exercised as to what part of the sunlight it was that acted upon a plate on which was a certain chemical compound, so that an image was obtained. They therefore experimented with various tinted glasses, thus cutting off different parts of the spectrum, or colour-band. Then they discovered that the most powerful part of the spectrum was invisible—was what we now call the "ultra violet" rays, and that the chemical action of sunlight lay solely in those colours marked above as "Finsen rays." You will now understand why photographers can work at their plates only where red light enters, because the red you see is quite at the opposite end of the spectrum to the violet or chemical rays.

Years went on, and experiments with light taught us the beautiful art of photography as it now exists, and taught us through spectrum analysis what the sun consists of, and the stars, and all sorts of wonderful things; but it was not till 1893 that Dr. Finsen, of Copenhagen, began to experiment as to whether these chemical rays of light could not be used as a therapeutical agent. He considered first the dark skin of the negro and those who live continuously in strong sunlight; he also considered the solar eczema of Alpine climbers and others who temporarily face strong sunlight. He painted part of his arm black, and exposed it for three hours to a very hot sun; the result was that, when the black was washed off, the part protected by it was unhurt, but the adjacent parts were red, and erythema, accompanied by pain and swelling, followed. Evidently the light had an inflammatory action on the skin. Dr. Finsen decided to try keeping certain small-pox patients in rooms to which red light only entered, and from which, therefore, the chemical, or inflammatory rays were excluded. The result was that not one of the patients had any suppuration, secondary fever, or scars, and to nurses who know how dangerous is the suppuration-period, this is strong evidence. Strangely enough, an old superstition has always existed in country districts in England in favour of red curtains, coverings, etc., in all feverish cases, and Dr. John Gaddesden, who died in 1361, is stated in his obituary notice to have "cured a son of Edward I., by red light, of small-pox."

Dr. Finsen's "Chemical Rays in Variola" was published in 1894; in 1895 he published another paper on "Light as a Stimulant," and described some experiments on eggs that showed that light provoked movements in the fœtus, and that this faculty must be specially attributed to the violet rays. Other experiments on living animals were carried on, and Finsen arrived at the conclusion that the solar energy not only gives us (1) light, (2) heat, but also a third chemical effect which promotes life and energy. So the old sun-bath theory received confirmation.

Then in 1897 Dr. Finsen published his remarkable paper on "The Treatment of Lupus Vulgaris by Concentrated Chemical Rays." The experiments described are founded not only on light as a stimulant, and its action on the skin, but also on light as a bactericide. That sunlight kills germs is a truth always impressed on nurses, and if any nurse has ever left her bacteriological cultures where sunlight could reach them, and returned and found them all dead, she will not need that this point should be further argued.

Every person in their youth has experimented with a lens known as a "burning-glass," and concentrated the sunlight on a spot on the hand till a burning sensation was felt, or on a piece of wood until the wood took fire. It is obvious that light could not be thus concentrated and merely supersede lupus by burns, but the heat of sunlight is in the red and yellow rays, and all the stimulating and germ-killing powers in the blue and violet rays. So at first Finsen filled his hollow "burning-glass" or lens with a solution of blue which cut off all the red rays. An ammoniacal solution of sulphate of copper was, in fact, used. The lens was made of quartz, which long ago was proved to allow the passage of the invisible rays much better than glass; and the lens was slung in a frame like that of a shaving-mirror, so that it could be turned any way or raised or lowered.

The patient was provided with a couch in the garden or on a veranda, the rays of the sun were focussed through the large lens on to the spot to be treated, an area of 1 to 3 centimetres in diameter being submitted to the chemical rays for at least two hours daily. But though living tissues are permeable to light, the red colour of the blood was found to be deleterious to the therapeutic action of the light. This was proved by putting a sensitive photographic film behind a patient's ear, and turning the violet rays on the ear—there was no action on the film for five minutes; but when the ear was pressed white between two plates of glass the film turned black in 20 seconds. Therefore the second lens for focussing the light was also made a pressure-instrument, and the special duty of the nurse was to keep this lens pressed to the spot so as to render the skin anæmic or bloodless.

Though Dr. Finsen distinctly states that the sun's rays are the best curative source of light, and that the outdoor séance greatly helps towards the patient's general return to health, the apparatus which generally bears his name, and which will be described in a subsequent article, is an artificial light. Of course, sunlight is seldom to be obtained in northern latitudes, and in cities such as London, and when it was first attempted in the grounds of the London Hospital, it was found that by the time the patient and the apparatus were ready the sun had generally retired again! But still there will probably be in the future a great opening for sun-treatment in more Southern lands, especially in out-of-the-way places where lamps are not fitted; or with rich folk who prefer to take a nurse to the South of France, to attending a hospital n London.

Also, at the bottom of the whole treatment lies again the great hygienic truth of the value of light; and if it only once more rouses the district nurse to plead for the removal of blinds, curtains, or flowers from the window, or drives home the fact that sunlight is the great germ destroyer, good will have been done.

*(To be continued.)*

### Hospital Economics.

By Anna Lowell Alline, Treasurer of the American Society of Superintendents of Training Schools for Nurses.

The course in Hospital Economics, Teachers' College, Columbia University, U.S.A., was established in 1899. Teachers' College, as the name implies, prepares teachers for advanced work. This, then, would seem to be the proper place for nurses having already completed their full course of training to take up such studies or course of study as would fit them for the responsible position of training others.

We all know too well that the nurse has by far the larger part of her time allotted to the practical part of her education; not only is there insufficient time for the theory, but even that small allowance is encroached upon very often by the practical duties which are ever calling for attention. What then can be more important than to spend a year in endeavouring to establish in the minds of nurses who intend taking charge of training schools that the theory is essential to the practice?

The "how," the "what," and the "when" are questions which come to the students; the first is well solved, for the second the student anyhow obtains the essential material to cull from, and as to the third from her larger point of view, she is better able to adjust her classes in the future to whatever condition she may find most suitable.

Special branches outlined in this curriculum are as follows:—

Psychology and methods of teaching: requiring three hours' class work a week and considerable reading and thought outside of class hours, observation of class work in other departments and papers written from time to time. This gives a good foundation for the proper method of conducting class work and developing the individuals of the class. When we think of class work in the training school, with nurses physically exhausted, and attempting to handle an important subject in such a way as to make at least some impression on their benumbed brains, we realise that the instructor must have all the previous preparation and equipment in the way of charts, etc., that is possible.

That part of the work more directly in line with our previous training is the Hospital and Training School Organisation and Management. Courses of lectures are given covering all the subjects to be considered in a thoroughly well-organised hospital and training school; small hospitals and special hospitals are also considered.

Discussions follow the lectures. In the practical work of this branch, topics are assigned the students and visits are made to various institutions. In the case of hospitals a close inspection is made of the general plan of building or buildings as to heating, ventilating, management of the whole and of departments, arrangement of ward with serving rooms, toilet rooms and equipment. The whole history of the nurse passes under their scrutiny, from her first application to her diploma; nothing seems too personal—neither character, education, previous to and during training, diet, dress, nor personal habits.

Practical talks by settlement workers and sanitary inspectors turn our thoughts to less familiar channels, but still not outside that unlimited number of subjects in which a superintendent must be posted.

Anatomy and physiology are taught with the direct aim in view of the presentation of these subjects to a class of pupil nurses in the training school. Text books and material, as the skeletons, models, etc., are examined and thoroughly discussed as to their use and value in the class room.

The other studies are like so many links in a chain. While each is distinct in itself, yet the combination makes a complete whole by the end of the year. This is speaking in a general way of the science which treats of food material for the human body. First is the cell life, both animal and vegetable, what it is, its growth and reproduction studied microscopically. In the next step the students spend not less than five hours a week carrying out a series of experiments in the chemical analysis of food material which enables them to recognise their composition and classify the nutritive ingredients. They subsequently go to the domestic science department and study quantities, method of mixture, and combinations necessary for the finished dish.

The final step is calculating dietaries and dietary standards. This alone is quite a hospital problem and requires careful scientific training to put one in a position to cope with the many dietary standards in a single institution. The economic question is not neglected in any part, and must be based on scientific cookery and science of nutrition.

So far, only the serious side of a year of hard study has been considered. The old adage of all work and no play would give the same results here as elsewhere. Far from this is the actual fact, however. The environment is most pleasant, the group of University buildings is grand, and is situated on high ground overlooking City Parks on the one hand and the Hudson River on the other. The museums and libraries are a great source of diversion as well as the greatest help in study hours. Concerts, entertainments, and many social functions, to say nothing of the clubs and gymnasium, furnish delightful recreation.

The advancement in hospital nursing has been marvellous in the last fifteen years; the course in economics is but one phase of it. The step is full of promise. It is the rational development of a systematic plan to meet the demand in the hospitals and training schools for instructors and more competent heads of departments.

My short sketch is a mere outline of the work actually done by the class. It is only intended as suggestive. The newness of such a work means constant change, and progress is the watchword.

### The Nurses of the Great Northern Central Hospital.

A CHAT WITH THE MATRON. BY OUR COMMISSIONER.

Though one of the smaller London Hospitals, the Great Northern Central enjoys the reputation of being admirably managed in all its departments, and it has at the head of its nursing staff a lady who has earned the respect and confidence of all who have been brought under the range of her influence. The association of Miss Mary Hull, the matron, dates from before the removal of the hospital to its present admirable site in Holloway Road. Trained at St. Thomas's, she was appointed in 1887, when the old hospital in Caledonian Road, consisting of five houses adapted for the purpose, was in existence, and has seen the development of the building into its present shape, with the round wards, which are its most striking feature. When I had been with the matron through several of the wards, I asked her about the training of the nurses at the time she joined the institution.

"We had then only one year's training," she replied; "but this soon after I came was altered to two years, and at the end of five years we adopted the three years' course, which has therefore now been in operation for ten years."

### The Sisters.

"What is the number of nurses at the present time?"

"Including sisters, 42. Counting the assistant matron there are 11 sisters, one in charge of each ward, a night sister, a sister for the operation theatre, and one for the surgery. So far as the rules for the sisters are concerned there is a certain amount of elasticity about them, but I like to keep the matter in my own hands. Their hours of duty are not quite the same as those of the staff nurses. I hope before long to arrange for them not to be in the wards until 8 o'clock in the morning. At present they are on duty earlier."

"And their salaries?"

"They begin at £30 and go up to £35. I also hope to get the salaries increased shortly. The sisters have a month's holiday in the year and a day off once a month, sometimes more. The night sister, if she has nothing to do, sits in the room close to the wards; and the night nurses, of whom there is one to each ward, are relieved, if possible, for half an hour by the extra nurse who is employed for the purpose of relief. I find that this arrangement is greatly appreciated, though it does not seem much. As to day duty, there are a sister, a staff nurse, and three probationers in every ward; a sister and a nurse for the surgery, and a sister and a nurse for the theatre. The out-patients' department, which is worked with the surgery, is quite one of the best in London."

### The Probationers' Fee.

"The staff nurses are, of course, probationers in their third year of training?"

"Yes. With regard to the age of probationers, we take them between 23 and 35. As a rule there are plenty of candidates. If they are accepted at the end of two months, they are required to pay a fee of £10."

"Do you find that the fee stands in the way of obtaining probationers?"

"On the contrary, the condition of a fee answers admirably. It might not answer so well in a large hospital, but this being a small one there is no difficulty. Our probationers are all drawn from much the same class, with the result that they get on very well together. The salaries have been increased during the last five years."

### Duty and Holidays.

"What do they receive now?"

"At the rate of £20 for the second year, and £25 for the third year. With regard to duty, the staff nurses and probationers are in the wards at 7. They have two hours off daily, and in addition half an hour off-duty is allowed after the morning work. Each staff nurse and probationer has a day's holiday, from 7 A.M. to 10 P.M. every month. As to the yearly holiday, a fortnight is given the first year, three weeks in the second, and a fortnight in the third."

"Why three weeks in the second year?"

"My experience is that in their second year the nurses are more tired, and want a longer holiday than at any other period of their training. In the third year they are not only less tired, but they know that at the end of it they can have as long a holiday as they choose."

"Have you ever thought of extending the term of training to four years?"

"In a hospital like this a fourth year is not advisable; and my own opinion is that at the end of the three years it is desirable they should enjoy a long holiday."

### The Hospital a Home.

"The House Committee," continued the matron, "prefer that if possible our staff nurses should become sisters when vacancies occur, though I think that it is better for them to go elsewhere for a time. One advantage is that they come back on a different footing, with rather more authority. Moreover, the further experience gives them wider views of life, and they see how things are done in other hospitals. But they are generally pleased to come home."

"Then they regard the hospital as a home?"

"I should be very sorry if they did not. The nursing staff here is exactly like a large family; we all know each other so well. There are no strangers. Another advantage, I think, is that there are no students. There is no play, but plenty of work."

### Practice and Theory.

"You have a large number of acute cases?"

"Most of the cases are acute. We get the patients off to convalescent homes as soon as they can be removed."

"What about the theoretical training?"

"Regular courses are given by members of the medical staff on medical, surgical, and obstetric nursing. A course of instruction is also given in each year on medical rubbing. The assistant matron also holds classes. No nurse receives a certificate of efficiency until she has passed an examination satisfactory to the examiner."

"Do your nurses get good appointments when they leave you?"

"Many prefer to go in for private nursing. But they always find it easy to obtain appointments. One of our nurses is now matron of Peterborough General Infirmary, another is head of a large plague hospital in Bombay, and others who were trained or were sisters here are respectively matron of Cornelia Hospital, Poole, a lady superintendent of the Herbert Hospital, matron of Ealing Cottage Hospital, and lady superintendent of the Merchant Taylors' Home for Ladies."

### The Question of Uniform.

"I suppose you supply uniform?"

"Indoor uniform. The staff nurses wear different caps from the probationers, and the sisters have a different dress. As to outdoor uniform, we do not provide it. And I do not see why hospital nurses should wear it Of course, in the army, where the nurses have a uniform of their own, it is another matter. But as nursemaids or anyone can wear the uniform of a hospital nurse, I think the latter should dispense with it out of doors."

Inadequate Accommodation.

"You are not well off in respect of accommodation for the nurses!"

"No, we should be very glad to have a nurses' home. But we are very poor, and the committee have to be extremely careful in their expenditure; and we want more space as well as more money before we can get the home. As you have seen, there is only a small sitting-room for the nurses; and in the single dining-room meals are going on all day. The hospital was modern when we started, but though we make the most of the room we have, we are now very cramped. We have just taken a temporary house to increase the accommodation for the nurses."

"They do not all have separate bedrooms?"

"No, unfortunately, that is impossible under existing circumstances. But many of them have a separate room, and we give each separate furniture and a screen. One reason why we are so particular as to the class of girls we receive as probationers is that some of them have to share rooms. No special recreation is provided, but I encourage the nurses to bring their cycles; and, for the rest, they get their amusements with their friends outside."

The Pay Wards.

"How has the addition of pay wards affected the nurses?"

"It has certainly not had any detrimental result, though we find the paying patient rather difficult to nurse. The pay wards were opened in 1896, and we have three rooms each with two beds and four rooms with a single bed. The utmost the public pay is two guineas a week. The wards are usually full."

"In what respect are the patients difficult to nurse?"

"They are apt to exaggerate their own illness. This is, perhaps, partly due to the fact that the rooms are necessarily much more dull than the ordinary wards. The committee are very particular about not taking in any patients who can afford to pay for nursing at home. Each case is most searchingly investigated, and no one is admitted without the sanction of the committee. If a case happens to be urgent, the patient is taken at first into a general ward. Many of the patients are clerks in banks and offices, or governesses suffering from typhoid, or requiring to be operated upon."

"Have you any religious difficulty?"

"No. The nurses attend any church or chapel they choose; and there is a service every Sunday in the wards, and one during the week."

Royal Visits.

"Do you like the surprise Royal visits?"

"So far as the nurses are concerned the surprise visits, which are, of course, informal, are much preferred to the State visits. The Prince of Wales, who is our President, and the Princess have been very good to us, as the King and Queen, who opened this hospital, were before them. But we are all very loyal, and Royal visits always excite enthusiasm."

### To Hospital Nurses About to Take up Private Work.

A PERSONAL LETTER.

Dear Nurse,—The fully-qualified probationer who exchanges hospital work for private nursing, whether on the staff of a home or "for her own hand," is somewhat in the position of a soldier detached from the ranks to maintain peace and order in a hamlet placed under martial law. No officer is at hand, no fellow soldier at his side. Those placed suddenly in his charge know nothing of military etiquette; words of command are to them an unknown tongue, and this sudden apparition in uniform, is a creature from whom, whatever may be hoped, not a little is also to be feared.

A Scene of Confusion.

Something of this, Nurse, will probably be the feeling towards you in the household of a "private case." Your hospital life has been passed in the midst of accident and disease; your associates have been doctors and nurses, familiar, like yourself, with sight and speech of sickness and death; the abnormal was to them and to you the normal. But remember this is not so with the people who live behind that door with the muffled knocker, and the "Please do not ring" card on the bell-pull. You are about to enter upon a state of things which, unless you have thought it over quietly, will appear perhaps inconceivable to you. In this house it is more than possible that illness is an unknown quantity, a new factor in the equation of life. There are, it is true, houses where disease is a visitor only too familiar; others where the unexpected is received with calmness and forethought. But such are rare. You are likely to enter on a scene of more or less confusion—probably more. The younger children, vaguely frightened at the alarm of others, clamour for "Mother," who if not herself the stricken one, is at the bedside. The older inmates wander about unable to settle to any occupation which will calm their minds. In the kitchen the servants are flustered, and meals and work generally are at sixes and sevens. Unless you have an inexhaustible fund of sympathy, such chaos arising out of one case of illness will seem almost ridiculous to you, after the clockwork routine of a hospital with its 500 occupied beds.

The Importance of Tact.

But I hope you did not forget your sympathy when you set out for your case, nor a good supply of tact; nor, above all, conscientiousness. So taking your tact in both hands you will set yourself to bring order and method out of confusion. Do not try to reduce the disorder of the sick-room to the severe primness of a hospital ward in the first five minutes of your arrival. A friendly soothing manner with both patient and friends will soon pave the way for any needful rearrangement. Set things to rights little by little, and not with an air suggestive of dissatisfaction with everything done before your arrival.

Outside Anxieties.

You must not expect that all your anxieties will lie within the sick-room; it is unlikely that you will be so fortunate. With the confidence of the hospital you may perhaps give an order for some supply of vital importance—milk, ice, or the like—to be ready for your patient's use during the night. Do not think that, your instructions once given, you can dismiss the matter from your mind till the thing asked for is actually needed. You must see that the ice has indeed been sent for; that the milk is really ready in the house.

The Ugly Duckling.

But, you say, all this suggests that I have come into a house full of incapables! Well, that is precisely what I want to prepare you for. If there is but one master-mind in the family you will soon discover your good fortune and find that there is someone upon whom you may always rely for boiling water; someone who will keep the doors from banging

### TO HOSPITAL NURSES ABOUT TO TAKE UP PRIVATE WORK—*Continued.*

he children from screaming, and who is always ready with whatever it is that is suddenly needed. This "someone" will probably not be the artistic or musical daughter; certainly not the spoilt beauty. Someone it will perhaps be who does not take a very prominent place at other times. It may be the "ugly duckling" of the house; perhaps a servant; perhaps—I have known it so—a charwoman. Whoever it is, you will discover her before long, be sure of that; for times of panic and disaster are those in which the ugly duckling puts on her swan plumage. It is upon her you will come to rely for punctual relief at your hours of rest; for the passage being kept quiet, and the coal-scuttle filled, and for the absence of those taps and inquiries at the sick-room door which some of us know only too well.

### Trust Your Help.

If you are so happy as to have such a helper, trust her and show that you trust her, but do not trust her needlessly. Remember that after all her willing help is but "unskilled labour." Do not crowd her memory with details when you leave her in charge during your rest or your walk; have a time-sheet drawn up for the medicines, stimulants, etc., which are to be given, with notes of anything else to be remembered. A hint that you find such an aid to memory useful yourself should remove from the most sensitive any suspicion of suggested inferiority.

### The Patient's Relations.

But you may have no such able coadjutor. In that case you will need all the tact you brought with you, and, by the bye, see that it is of the very best quality. Otherwise, how will you manage all those with whom you come in contact? the fond wife, mother or sister, already jealous of the doctor's cruel order that she must relinquish the nursing of her dear one to a stranger; the servants, doubtful how to treat the wearer of a cap and print dress, and keenly on the look out for "airs;" the host of relatives and friends, each so confident of "dear so-and-so's wish to see me just for two minutes?" The doctor will, and in extreme cases must, come to your aid in enforcing needful orders and restrictions. But he will be better pleased if you can manage matters without an appeal to him at every visit, and moreover your own position in the household will be infinitely more pleasant. Those about you have a claim on your forbearance and consideration second only to that of the patient. Put yourself in their place, try to realise their feelings. Tortured with anxious love, restlessly eager to be doing something for their dear one's life and safety, they must yet stand aside idle, or at best act under the orders of this calm impassive stranger, to whom their loved one is but a "case."

### The Unforgivable Sin.

Perfect sympathy we cannot all feel; boundless tact we do not all possess; and failing these we must foster and improve what little of each we are vouchsafed. But conscience is a universal possession, and, in nursing, a lapse from the highest conscientiousness should be the unforgivable sin. In private nursing there is more temptation to carelessness; to think "it doesn't matter" because you are where you will perhaps not be found out, where there is no lynx-eyed matron or "sister," no jealous fellow-nurses. The patient and the friends may laud your sympathy and tact to the skies. What do they know of your courting death by putting the antiseptic gauze away without its sterilised wrapping? of your using a sponge, or handing the surgeon a probe, which has dropped on the floor since its sterilisation? You are here upon honour; your conscience is your sole accuser. Take, then, what little sympathy and tact you have, and make the best of them. But unless you carry with you a conscience of superfine quality, and can be sure of keeping it in perfect working order, then I say emphatically the nursing profession is not for you. Yours,

"TAUGHT BY EXPERIENCE."

---

### The Routine of a Cottage Hospital.

BY A WORKING MATRON.

I suppose that from the time when nurses were probationers, changing wards, and later on when their training ended, they change hospitals or take up another sphere of work, they never quite lose a certain nervousness as to what the future holds in store—whether they will be equal to the emergencies which may arise, or fulfil their duties to the satisfaction of those in authority. So, though I had been head nurse in one of the smartest surgical wards in a large London hospital, where we prided ourselves on our up-to-date aseptic methods—though what surgical ward does not?—and I had gained the L.O.S., when the matron was good enough to procure me the charge of a wee hospital of seven beds, I thought my cup of happiness was full, but I was proportionately nervous.

### My Arrival.

It was not at all nice saying "Good-bye" to the old hospital and travelling 200 miles north. Later upon arriving at a small station on a dark night I realised that the feeling like a hole in my heart was homesickness. This place, which I now remember I ought to call a town, but which is not much more than a village, is economical in its expenditure of gas, and no lamps are lighted on moonlight nights. Unfortunately, on the night I arrived it was cloudy and no moon was visible; consequently I stumbled over unpaved streets in a most disheartening manner, and I do not think I should ever have found my way if a kindly labourer had not led me to the very gate. Even then in my effort to find the front door I fell over a kennel, waking a huge St. Bernard dog, who barked with such gusto that the door was opened immediately. At first I was charmed with the place, which is picturesque in the extreme. I found it in charge of a nurse from a nursing home, who made me very welcome, and who told me that she had had the hospital under her care during the interval between the last matron's departure and my arrival. She seemed to have managed very nicely, but confessed that she did not know much about the usual arrangements.

### Going Round the Hospital.

I unpacked that night and slept in the prettiest spare room imaginable, and the next morning we went round the hospital. To begin with, I was informed that the servant was new, having only arrived the previous day. When nurse said to me, "It is so nice for you to have a fresh maid, you can get her into your own ways," I thought inwardly that I should infinitely have preferred someone who knew her work and the methods of the place, but I said nothing. Silence is my principal form of discretion. We visited the surgery, which is also the theatre, and it was here that my heart sank lowest. The wooden operating-table, with legs like an elephant's; the boarded floor; the kidney-shaped dressing dishes, of some unboilable material; and last, but not least the steriliser, which was not big enough to hold the basins! It was seated on a methylated-spirit stove, which consumed sixpennyworth of spirit before the water would boil. Finally, as the finishing touch, I was introduced to a very unprofessional wicker chair as the most convenient one in the hospital in which to extract teeth. In a faint voice I said it was very nice, and we passed on to a ward. It was quite charming; beautiful engravings, lovely chintz curtains, a Turkey rug, a richly tiled fireplace, and three patients, who glared at me in a semi-hostile manner. I found that they had all been fond of the previous matron, very happy with the locum, and had decided that a third could never come up to the other two. Subsequently, we were very good friends, and when nurse had departed they were most energetic in describing my duties to me.

### Night Nursing.

My bedroom is situated between the two wards, with curtain-covered windows overlooking them. Nurse explained that I might leave the windows open—particularly if there was a bad case—as I should hear the patients' bells. Here was more food for thought. How could I possibly sleep, even supposing there was only a convalescent patient next door? Had I not just come from a hospital where a ward full of snorers had its night nurse, who is relieved for the supper half-hour, in order that the ward may not be left a minute? Yet here I was told that I might go to bed and trust to hearing the bells. "No, indeed," I said to myself, "come what may I will make a half-hourly tour of inspection always." However, by this time I have learnt to manage differently. Still, if I am in the least anxious about a case, I cannot sleep, but sit up till the patient is better, or till I am obliged to get a nurse. But it is wonderful how one can arrange for patients to manage in the night without calling for help too often, and how well they will sleep without the constant supervision of a nurse.

### The Trials of a Housekeeper.

At length the time came for the nice locum to depart. I felt as if I were losing my sheet-anchor as I waved my hand at the door and turned round to find the new maid, at my side. "Please, sister, what will you have for dinner?" Goodness me! Memories of the alternate boiled mutton, roast beef, roast mutton, or boiled beef floated through my mind. Then came the reflection that we couldn't have a fresh joint every day for six in family, and I sought inspiration in the larder. Here I found remains enough to constitute a family meal, and re-assured myself that there was nothing to send me into an inward fume; though for a Londoner, where all things can be procured any day, it is a little disconcerting at first to find that beef can only be purchased on a Friday, and mutton on a Tuesday.

### First Aid to Out-Patients.

My equanimity was next disturbed by the maid telling me that a man in the surgery wished to see me. "A loomp on 't shoulder" was the trouble. I looked, felt, prodded, questioned, and finally discovering that he lived near, I told him to call at a time when I knew the doctor would be here. That matter was certainly disposed of, but I could not help thinking that there might be times when the patients would come from a distance, needing instant relief, and the doctors might both be away. And truly there have been times when I have been extremely puzzled what to do with out-patients with their queer unexplainable symptoms, and have felt that an abdominal section in difficulties would be infinitely easier to manage. But one very soon learns simple remedies which may be applied by a nurse to ease the patient till the doctor prescribes. Here, however, I may say that whilst a cottage hospital is an excellent place in which to obtain confidence, it should be equally good in teaching caution and impressing the fact that one must never be tempted into prescribing drugs nor treating medical patients. I say medical, for cuts, wounds, and poisoned fingers must sometimes be treated by the nurse unless she would always be sending for the doctor. As for bad legs, usually ulcerated, I did not know there were as many in all England.

### Economy in Dressings.

At first I was very liberal with the dressings, but it was not long before I found how soon they mounted up, and that my surgery bill was larger than it should be. I spent some little time in perusing chemists' catalogues and comparing the prices of lotions and the different qualities of lint and wool, in the knowledge of which I was quite at sea. Then I remembered that, though cautioned by my various ward sisters as to economy and carefulness in the use of certain dressings, etc., I had never learnt practically how expensive they were, and recalled, with some compunction, how we nurses used to grumble at a sister whom we considered miserly in the way she counted out pieces of lint and doled out bandages. I can account for it now; she had been matron of a cottage hospital!

### Infected Bedding.

The last shock I experienced was the worst. When I arrived there was a convalescent patient who had been suffering from a perineal abscess. Pus had poured from it, but it was now nearly healed, and the man was ready to be discharged. I asked the doctors where I could have the bedding baked. I was told that there was no arrangement for the disinfection of mattresses, consequently a clean surgical case had to take its chance. I do not know what I should have done if I had not discovered that the workhouse had lately built some ovens for the purpose of baking the tramps' clothes, and the master was good enough to allow my mattresses to be done in that way. I thought to myself at first how nice it sounded to be called the "matron of a hospital," but when I scrubbed my macintoshes, cleaned out the dressing pail, and surveyed the surgery "brights," I felt that there was quite as much probationer as matron about the post. Later, when the servant fell ill, I became more of a cook than anything else. However, it is all most excellent practice. Since I have been here there have been times of enforced idleness, but a couple of patients or even only one bad case soon changes all that, and perhaps for two or three weeks there is no getting outside the door, for there are not even relatives to take one's place here as in private nursing.

### The Cook's Duties.

At one time I had a stout and helpless patient with a great tendency to bedsores, and my ingenuity and skill were much taxed to move her about. I was obliged to have recourse occasionally to the servant. Shortly afterwards she left, and one young woman who applied for the situation wished to know whether "there was much blood to wash up; and should she have to nurse men as well as to cook?" I gently explained there was no nursing to be done, as I was hiring a cook and not a nurse. But I found wild tales had been spread about as to what "sarvents did at t' 'ospital," simply because I had asked help to lift a patient! With all its difficulties it has been a delightful eighteen months; and though I am returning to the busy life of a big place, I doubt whether I shall be happier than at my little Cottage Hospital.

### A Recollection of Easter Monday.

BY A HOSPITAL SISTER.

It was three o'clock in the morning when the surgery bell gave one small sound. The nurse turned up the gas before opening the door, and the sight that met her eyes was pathetic in the extreme. A tiny girl of about seven years old, with a little pale wan face, large black eyes, black hair, and clad in a thin black frock, was holding in her arms a small baby boy, apparently about eighteen months old.

She gasped out, "Sister, take him—quick—his cough's that bad, and his breathen's awful; he's coughed all night and I can't abide to hear him."

The staff nurse took the boy from her and led the little girl in. She saw in a moment that the baby was desperately ill, and at once removed some of the many wrappings which were nearly suffocating him.

"His name is Billy West, and I'm Miriam; we live in X Buildings, Rahere Street; and I've carried him all the way. "Mother's dead," she said, in answer to the staff nurse's inquiries. "She told me to take care of Billy, and he's got terrible bad since mother died, so I'd got to bring him; but father don't know I've come, 'cos he drinks and he's not come home." Then, with the tears streaming down her poor little white face, she looked hard at the nurse, as though weighing her trustworthiness to take care of Billy. She glanced at the silver cross on her watch chain—

"Be you a Catholic, sister?" And when nurse told her she was not, she said, "Billy's a Catholic, them's his beads;" and her small dirty hand placed a little much-worn rosary in the nurse's hand.

"Give Miriam a kiss, Billy," she said, bending over him like a little mother, but poor baby, he was quite unconscious of it all, and she turned away and was down the steps before nurse could say anything to comfort her. Billy was carried upstairs to the night nurse in charge of the children's ward. She washed him by a big fire, and then placed him in a cot in the warmest corner of the ward. Cosy curtains were drawn round, and a steam-kettle put by the side of the cot. A doctor was called up who examined Billy, and his name was sent down in the morning and put upon the "danger list." At seven o'clock a gentle tap was heard at the door and the same little figure appeared who had come to the surgery in the earlier hours of the morning. She had not been to bed, she said, and father had not come home—could she see Billy? It was all spoken in a hoarse whispering voice, and nurse saw in a moment that she was quite exhausted, and that she shivered and coughed as she spoke.

When she reached the cot her loving eyes seemed quickly to detect some change in Billy, and she turned her pathetic little face to the nurse in mute inquiry. "Yes, dear, Billy is very ill. Would you like to kiss him?" In an instant the tears rolled down from the lovely black eyes, and in a hoarse voice she said, "I must go and find father." She put her hand over the side of the cot and took hold of the white fingers, kissed them, and whispered, "If you go to mother 'fore I'm back, Billy, tell her I cared for you and carried you all the way." She left the ward on tip-toe, as though alarmed at the noise her heavy boots seemed to make on the polished boards, and nurse did not notice her go. When I went my round to each bed with the night nurse I looked with alarm at the new little occupant of the cot by the fire. I inquired what relatives the baby had, and was told of the devoted little sister who had gone "to find father." The day wore away, and it was 7 o'clock before Miriam again appeared at the door. It had rained from morning until night, and the poor little soul was drenched through. She must have tramped the streets all day. Her face was colourless, and she seemed utterly worn out. She tried to speak, but no voice would come. I raised her in my arms, undressed her, and laid her in a bed next to her little brother. Her large dark eyes were wide open, but she seemed scarcely conscious.

. . . . . . . . . .

Five hours later William West was standing between the beds, looking down upon his two children. I had gone to X Buildings, in Rahere Street, and, after mounting many flights of steps, found the right room. At the door I met William West coming out. The poor man seemed distraught. I told him in a few words about his children, and he said he had been searching all day. He had got to his home at 10 o'clock that morning, had been told by the woman below that Miriam had taken the baby to the hospital, and afterwards had gone to his old haunts to find him, but no one seemed to know to which hospital she had gone. He told me that he had had regular work until a fortnight ago, when his wife died. He had then broken down, and had been drinking ever since, until that day. The man seemed wholly sobered by the shock. I led him to the cot where little Billy lay dying. He bent down and took the tiny fingers in his, and found there his wife's rosary, which she had put on her baby's neck the day she died.

"May God forgive me, Mary," he said, in a broken voice, "if I have killed both your children." The baby died that night—but Miriam was spared to him. Night after night he came home from his work and sat by his little girl's bed. Ten days passed before we could tell him she was out of danger. One night she seemed to know him and hear his voice, when he said "Miriam, speak to your father, lass, look up and speak to me, my lass"—a weak little voice answered "Better soon, father." He knelt by her bed and whispered some words to her while he passed his hands through her curly black hair—it had been something which brought a smile to her lips—and the man's face was radiant as he turned to me and said "She'll do now, sister. She'll do. She remembers her dad's old joke about her hair. I'll take my little lass home yet."

She very slowly recovered, and the day came at last, when she was able to understand about Billy; I shall never forget the look that came over her sweet thoughtful face. She was quite quiet for a moment, and then said "It is all right, sister, for you see mother's got Billy and father's got me." Unselfish from first to last was this little maid.

The day her father came to take her away from us was Easter Monday, and it chanced to be her birthday. When he stood by the door I could not help noticing what a strong, steady, manly looking fellow he was, and hesitated whether to speak a few parting words to him, when giving up my little charge, but he forestalled me. "Sister," he said, "if all be well, I'll come every Easter Monday and bring my little lass to see you, and you shall judge for yourself if I don't mean well by her."

. . . . . . . . . .

He kept his word. Eight years I have been sister of this ward, and to-day it is again Easter Monday, but this time she came alone. "Father is married," she said, "and I am going to my first place."

She seemed all smiles and sunshine and full of hope. She brought me her photograph, and I have placed it on my mantelpiece amongst all my other hospital treasures—tiny gifts from "grateful patients." I often look at them and feel I could not part with one; they are full of memories and mean so much to me.

"The world is wide—these things are small—
They may be nothing—but they are all."

### Nursing in Danish Hospitals.

BY NURSE OLGA MÜLLER.

I have often been asked about nursing in the Danish hospitals. I trained at the Royal Frederick Hospital in Dr. Schous' Nurses' Home, Copenhagen. There the medical and surgical departments are in separate buildings, quite isolated from each other. There are about 100 patients in each department, with one head doctor, four candidates, a matron, six charge nurses, and 18 probationers. Other women are employed to do the cleaning. Every ward contains 12 or 14 patients. The walls are all painted, the floor brown (varnished), and it is washed morning and evening to make the air as fresh as possible. The shut-up stoves diffuse a regular heat night and day. Plants and flowers are not allowed in Danish wards, because I have heard doctors say that they make the air heavy and gather bacilli. At 8 o'clock the wards are ready for the doctor's rounds, which always occur between 8 or 10 in the morning, and between 7 and 8 in the evening.

### Patients in the Medical Ward.

The patients beds are drawn out into the wards away from the walls, so that the nurse is able to go all round the bed without difficulty. Most of the beds are about 20 inches high and are of iron, because these are cleanest. The bottoms of the beds are woven of wire, and spring a little, but not enough to make the patient nervous. In our hospitals the pillows are filled with wool, and the mattresses are stuffed with hair. These, however, instead of being in one piece are divided into three, so that the different parts of the mattress can be changed frequently. The nurse must be careful that the centre part is not hollow. It is very difficult to make beds for people who are crippled or hump backed. I usually arrange the pillow with a depression in the middle, then with my hands I shape out a ring in order that the affected part may rest properly and without pressure. If your patient is very sensitive never let him see you take the least notice of his spine. You will soon observe by your patient's countenance if he is comfortable or not. Those people who have a vocation for the work see and understand by the expression of their patient every little thing, and are able to give help when needed. By the patient's bed a little table is placed. This stands on the left-hand side, because if it were on the right the nurse would not be able to attend on the patient so well. Beside each bed is a towel, sponge and tooth-brush. All phthisis patients are isolated from the medical patients in a building by themselves, and all are given the open-air treatment. If the disease is only slight and it is taken in time the patient often gets better (more particularly if it is not in the family). In the Earsuns Hospital at Copenhagen a great many of the phthisis cases are cured. The patients have a tepid sponge bath morning and evening; they live most of the day in the fresh air and sleep in rooms with open windows; if very mild at night they sleep out in the garden in tents. These people live on the best of food and take four or five meals a day.

### The Surgical Wards.

There are only six patients in each surgical ward. The first week after an important operation the patient occupies a small ward alone. The operation-room stands on one of the landings, isolated from all the wards. It is a very large room with electric lights and the temperature about 65° to 70°. The floor is marble, and after each operation the floor is swilled out with carbolic (1–20); pipes are laid all round to let the water out. The operation-tables are white enamelled, and the pillows white oil-cloth; they are washed and carbolised before every operation and a sterilised sheet is spread over. Nothing is allowed which cannot be washed with soap and water or carbolic. Instrument tables are painted white with glass plates over. These are washed with spirit. There is also an ante-room, where patients are chloroformed before they are carried into the operation-room. In this room is the instrument and bandage closet. The doctor, matron, and nurses present always bathe before an operation. The doctor's jacket and matron and nurses' dresses are sterilised before being worn. A Danish nurse wears a white dress and short sleeves. If possible the patient comes into the hospital a week before the operation and bathes every day by the doctor's orders. In Denmark, if a patient dies in one of the large wards, the rules are for the beds to be screened round and the body not to be moved to the mortuary until after the lapse of six hours. The nurse must keep a window open beside the bed.

### The Reward of Duty.

It has been suggested with regard to the work of the nurses in South Africa during the war that it is ridiculous to call it "self-sacrifice" or "devotion to country;" it was only sham patriotism, half balderdash.—(*See Weekly Paper.*)

In Britain's cause her sons poured forth
  Their hearts, their wealth, their lives.
To Britain's faithful care they left
  Their children, homes and wives.

'Twas but bare duty, nothing more;
  Thank God! we own that, still.
O'er all the Empire, each one strove
  His sacred trust to fill.

And "Those at Home" in ev'ry land
  Tho' mourning, bravely cheer'd
And succour'd sorrowing ones, nor yet
  At others' service sneer'd.

And only we, sent out to nurse,
  Have met with bitter scorn;
And venom'd jibe and cruel jest
  From "Those at Home" we've borne.

We "madly rushed" out to "The Front"
  Our selfish ends to gain,
What did *we* care for death or wounds
  If others bore the pain!

"Excitement" "flirting" junketings"
  Devotion? "Balderdash!"
A Nursing Sister with a heart!
  Faugh! Sentimental trash!

Oh fellow-nurses! curb your tongues
  And stay the ready pen,
If you'd been sent, instead of us,
  How chang'd the story—then.

Not yours to give, is our reward,
  Nor yours to take away—
'Twas read in dying eyes, 'tis shrined
  In living hearts, for aye.

Some, over-grateful, roused your ire
  And called us "Angels blest,"
We only ask you to believe,
  Most of us did our best.

*A. N. S. B.*

### Hospital Balcony Decoration.

BY AN IRISH CORRESPONDENT.

The nineteenth and twentieth centuries have brought many improvements, not the least being the improvements to our hospitals, for now there is hardly a modern hospital which has not a balcony, which with a little care and trouble may be made quite a garden of flowers during the summer months. These balconies are a great joy to the happy convalescent patients, because there they can sit out and breathe in the fresh air to strengthen their weak bodies, and find plenty of food for reflection amongst the flowers, being brought face to face with nature in its purest sense. The floral gardens are usually the care of the sisters and probationers, and it is for them that I pen these few words, hoping that they may be found useful to some and encouraging to others. The flowers for this purpose must be of a certain class and kind; for instance, they must be cheap, inexpensive seeds to buy, for generally it falls upon the staff to purchase them, and as a rule nurses are not to be classed amongst the wealthy; while, secondly, annuals and half-hardy annuals are better than perennial, with some exceptions, of course, for gardening upon balconies is principally summer work. Perennials do not do so well, because in pots or boxes a great depth of soil cannot be given, which, though sufficient for one summer, would not supply enough plant food for all the year round. Many of the choicest flowers, however, will do best if sown in pans, boxes, or pots in the wards, and planted out afterwards into other boxes before being placed upon the balcony. All half-hardy annuals should be treated thus, and a great deal of amusement will be given in this way to the patients who can watch them taking their first peep into the light, note their growth, the making of their leaves, etc.

### Practical Hints.

They should be sown as follows, and the best time for sowing is the end of April or beginning of May so as to give them as long a period of growth as possible to insure a vigorous plant before the season of flowering; though it is safer as a rule to wait for full sunshine and daylight in order to keep up a continuous growth. The seed for box and pot should be filled with the best soil obtainable which should be made fine, and have some sand, if possible, mixed through it to render the texture porous. The seed should be then sown thinly over the surface, covered slightly, and well watered with a can with a fine seed rose upon it, then some squares of glass placed over the top and shaded with a piece of paper, for germination always takes place best in darkness. Should watering after this become necessary, take care to do it very gently so as not to wash the seeds out of the soil. When the seeds appear, remove the glass and stand in the full light but not in a draught, in a place, however, where the plants can get plenty of air, as if they do not get air they will be sickly, puny things, hardly worth planting out. If, unfortunately, the ward is a very cold room facing North or East, it would be folly to sow until April, so no definite time can be laid down for sowing. Care also must be taken not to plant out too soon if the weather should prove unfavourable, as it is better to wait a few days than put them out at the mercies of harsh elements. If, however, they cannot be sown indoors then half-hardy annuals cannot be used, and the annuals should not be sown in the boxes or pots upon the balconies until early in May, filling the seed-bed with as good a soil as possible and sowing very thinly, for if they are not sown thinly they will have to be thinned by hand later on to allow them enough room to flower. For balcony decoration there are two points of effect which one aims to secure, *i.e.*, the hanging-over effect of the plants as looked upon from the ground, and the effect obtained when standing upon the balcony itself.

The following are a list of plants suitable for these purposes:—

*Annuals.*—Candytuft, Gypsophila elegans, linum, lobelia, love lies bleeding, lupinus, mignonette, myosotis (forget-me-not), nasturtium, nemophila, poppies, schizanthus, Statice (suworwi variety), sweet pea, sweet sultan, Poa alpina (pot grass), paspalium elegans.

*Half-hardy Annuals.*—Ageratum, Tropæolum canariensis (canary creeper), nicotiana, nierembergia, nigella (love-in-a-mist), Phacelia campanularia.

I have said that perennials, though not so good as annuals, could be used, but of course they must be taken in during the winter and housed somewhere. The following are a few which are useful for this form of work:—Tropæolum speciosum, Tropæolum pentaphyllum, musk, statice, forget-me-not (kept on from year to year), pansies, Dielytra spectabilis, corydalis, geraniums, ivy geranium, aubrietia, arabis (single and double), creeping jennie, etc., etc. These perennials will be found most useful if first plunged, in their pots, into the boxes or stood out; and what could be more graceful than the ivy-leaf geranium with its fresh green leaves and its bright pink or blush-red flowers? If these few directions are carried out, and a little care and attention given afterwards as regards watering and picking of the dead leaves, the balconies will be well furnished in quite a little time, giving a brightness to the whole surroundings and a feeling of hope and new life to the suffering, who while they gaze at these glories of nature must feel, as Milton says of Nature—

"She has done her part, do thou but thine."

### A Morning with a London Parish Nurse.

This is a very thickly-populated parish, but so compact that distance hardly counts. We start out at 9 o'clock; our first visit is to a woman who lives with her two sisters on the second floor of a warehouse. They are shirtmakers, and have worked always for one firm. The rooms are fairly large and comfortably furnished; the patient had an accident to her knee and neglected it, so that inflammation spread from ankle to thigh. She has been in bed six weeks. There are several wounds to be syringed and dressed and her bed to be made, and three quarters of an hour have slipped by when nurse leaves, promising the usual evening visit. Now we enter a dirty court and climb two flights of narrow stairs, but the room we enter is beautifully clean. Here is a woman of 65 years to be washed, lifted on to a chair and her bed made. She is quite helpless through rheumatism, and has been bed-ridden over 15 years; her old husband is most useful and helps nurse beautifully. Next we pass on to a court larger and cleaner than the last, to see a little girl with suppurating glands. The front door opens into a very small room and a tiny staircase leads to another equally small; here live father, mother and eight little ones, all under 12 years old. The five weeks baby is safely tucked away in a recess over a cupboard. Everything is so clean and tidy that we wonder how the woman manages; little Nellie is doing well, so we change the fomentation and settle her again on her couch of two chairs. Our next visit is to two little boys—one has had croup and the other pneumonia—both are now convalescent, though still kept in bed. We find the mother washing them. It is a sad home, for father has been out of work many weeks, and there are six little ones. We pass on to a baby recovering from pneumonia, but find her fast asleep, hugging her rattle; she has been very ill, and still looks pale, lying in her big bed which is scrupulously clean. We make a break now for lunch, and then start again, up three flights of stairs, to a woman who has to be washed and dressed, for she is disabled by chronic rheumatism; a neighbour will make her bed, so we leave it stripped to air. Then comes a new case in a court—a delicate-looking woman with several boys; she is often ill, and now has a sore throat and high temperature, so Nurse makes her comfortable, gives her a gargle, and tells her to have the dispensary doctor. On our round we have had a message to go to a shop, where we find a woman who has just been prematurely confined: the baby was dead. A douche has to be given, the patient made clean and comfortable and a binder put on, and Nurse promises to call again in the evening. Then we hurry off to our last case on the fourth floor of tenement buildings. Here a little child with bronchitis has to be put into a pneumonia jacket, and while Nurse tacks the wool to an outer casing she has brought with her, the mother rubs Tommy with camphorated oil, and he is soon settled in bed. On our way down we are stopped many times, for Nurse knows most of the women, and each expects a kindly word. She has promised, if I will come another day, to show me how these, our less-favoured sisters, live, for a great deal of the factory work is done at home.

### Novelties for Nurses.

By Our Shopping Correspondent.

### MESSRS. EGERTON BURNETT, LIMITED. Wellington, Somerset.

I have once again received a box of patterns from Messrs. Egerton Burnett, Wellington, Somerset, who always have something new and attractive to show with the change of seasons. Their speciality, as my readers know, is the Royal Serge in various qualities and colours, but they have also a large variety of materials, both washing and woollen, suitable for uniform as well as private wear, and among the latter I should like specially to mention the Winceys and Shrinknaughts. The first is suitable for under-clothing, blouses, toilet-jackets, nightdresses, etc., and the great advantage is that it "does not get hard in washing." It is made in white, pink, blue, scarlet, and other colours, and the width is 31 inches, while the price is 1s. 9½d. per yard. The Shrinknaught is a thicker material, and is also a washing fabric suitable for the above purposes. It is a little cheaper, being 1s. 7½d., and the width is the same. It is made in stripes and in checks, in all the principal colours.

Besides these, there is a variety of pretty materials for summer blouses and dresses; among them the new mercerised linen is conspicuous, and, for "the nurse in hot climates," there are dainty white linens, drills and printed muslins. The "Aden," a canvas with a silky texture, is particularly fascinating. These, of course, are equally suitable for our English climate if we are fortunate enough to have real summer weather this year. Cottons and lawns, very pretty in colour and pattern, are extremely cheap, being only a few pence per yard. Then for coats and skirts, cycling costumes, etc., there is a great variety to choose from, from rough hairy materials to fine covert coatings, any of which would look well made up. The "Kildonan," a superior costume tweed, and the "Merrywood," a soft hairy material, are good examples of the rough class; while the "Enfield" (price 3s. 3d., width 49 inches), and the various beiges are excellent if one is wanting a neat costume of fine material. The "Bideford" is a similar material, and is made in pretty colours.

The number and variety of materials suitable for uniforms are very great. Many hospital nurses, having no choice in the matter, are saved a good deal of time and trouble, but for those whose circumstances leave them free to order what they like, I may mention that this firm always have good things at reasonable prices. Their drills and cottons are good and strong, and calculated to resist hard wear and constant washings, which destroy clothes as much as the actual wearing of them. The serges for outdoor uniform are equally good, and are specially waterproofed for nurses' use. It is hardly necessary to add that cloaks may be made to order by sending careful self-measurement on the forms supplied for the purpose. The same applies to coats, walking skirts, and cycling costumes. The linen collars supplied by this firm are 6¾d. each, and cuffs are 9¾d. a pair. A dainty calendar blotting-case for the year is enclosed with the patterns.

### MR. C. WILLIAMSON, 91 Edgware Road, London, W

I am pleased to call attention to some excellent and suitable materials for nurses' dresses, uniform or otherwise, sold by Mr. Christopher Williamson, of 91 Edgware Road. The Wynburg regatta is quite an ideal fabric for a washing dress for nurses. It possesses all the qualities essential, as it is most durable, has a very superior appearance, and is made in colours both pleasing and suitable. For hard wear and excellence of effect it would be difficult to beat this material, which is also very inexpensive. Another material which takes my fancy is a hair-cord gingham. This is finer and softer than the regatta, though very durable. It is also cooler. The colours are pretty and deep in tone. For summer wear the coloured cambrics will be found soft and cool. Mr. Williamson makes a speciality of cotton materials, and has so extensive a range that nurses are sure to find something suitable to their needs by visiting his establishment or sending for patterns.

### TRAVEL NOTES AND QUERIES.

Rest Home at Cromer (G. S.)—I do not know the place personally, but I have good accounts of it. Terms from £1 1s. if two friends share room. Single room £1 5s. There are also arrangements with increased terms for convalescents. Send me a stamped addressed envelope and I will give you the address. In applying use my name.

Seaside Place in Belgium (Sage-femme).—Write to the Hôtel de Bruges, Knokke, Belgium. Living very cheap, from about £1 per week. Close to the sea. Good excursions. Full particulars in The Hospital for September 1st, 1900. No, rooms would be much dearer.

Where to Break the Journey (Sister Maud).—You will have seen my second answer ere this. If you are good travellers it is unnecessary to stop anywhere. You leave London at 10 a.m., reach Paris 6.5 p.m.; do not take a *fiacre*, but go by the Ceinture Railway to the Paris-Lyons station. Leave there at 7.25 p.m., reach Neuchâtel 8.12 the next morning. Take food with you in a tea basket, enough for a light meal soon after leaving Calais and a substantial one after leaving the Paris-Lyons station. Veal and ham pies, fresh tomatoes, rolls without butter, and hard-boiled eggs are suitable viands. If the weather is not very hot make your tea beforehand with plenty of milk, and only needing to be warmed, if very hot you must omit the milk. Names of hotels—Neuchâtel Grand Hôtel du Lac (ask for rooms always on third or fourth floor), Grandson Hôtel Croix Rouge, Interlaken Hôtel du Lac or Hôtel Oberland, Meiringen Hôtel de l'Ours, or Hôtel Brunig.

Engelberg or Gersau (Susan).—Certainly I should prefer Gersau in your case. You may stay as long as seven or eight weeks for the sum you name. Second class return ticket *viâ* Brussels and Strasbourg, £5 10s. Pension at Hôtel Beau-Séjour or Hôtel Seehof from 5 francs. Church such as you wish I believe. The steamers are most convenient, and you will never be tired of walking on the Axenstrasse. Brunnen is not quite so suitable, I think, and is a little dearer, but both places are charming. Hotel at Brunnen Pension Hirsch, terms from 6 francs.

### Everybody's Opinion.

[Correspondence on all subjects is invited, but we cannot in any way be responsible for the opinions expressed by our correspondents. No communication can be entertained if the name and address of the correspondent are not given as a guarantee of good faith, but not necessarily for publication. All correspondents should write on one side of the paper only.]

### INTERFERENCE OF THE WORKHOUSE MASTER.

"Mrs. A. Richmond, Matron, Luton Workhouse," writes: In your issue of April 4th "A. F. C." complains of the interference of the workhouse master. "She thinks that the Guardians are aware of the state of affairs." I should advise her to satisfy herself on this point by stating her complaints to them in a straightforward manner, and if she gets no redress, put her complaints down in writing and forward them to the Local Government Board. They would receive the attention due to them, and she would then have the satisfaction of securing far more good to the infirmary than can be obtained by writing anonymous letters to the papers. *Nom de plumes* are all very well in their way, but one always feels inclined to doubt the genuineness of such charges or the sincerity of purpose of the persons making them when they have not the moral courage to append their names to their communications.

### THE APOTHECARIES' HALL CERTIFICATE.

"A Lady Dispenser" writes: As I see that one of your readers asks me to state how to qualify as a dispenser (Apothecaries' Hall) I give the following particulars. Six months' study is necessary, taken at any one of the London or provincial colleges of pharmacy. There are three subjects to work up—materia medica, chemistry (elementary), and practical dispensing. Either of these subjects can be passed separately—that is, if a candidate fails in one or two, but is successful in the other, she is credited with it, and has only the remaining subjects to try for again. The fees amount to about 15 guineas inclusive, but, of course, if the student is not living at home, board and lodging make it much more expensive.

### TO PREVENT DRAW-SHEETS FROM WRINKLING.

"R. B." writes:—To nurses in charge of bed-ridden and helpless patients, I beg to offer a suggestion for preventing draw-sheets from wrinkling. Have your draw-sheet slightly wider than the mattress, with a wide hem on either side through which run a firm wooden lath, such as is commonly used for window blinds. These laths have holes bored in them at each end, with corresponding button holes in the hem of the sheet. Pass through the holes strings of stout tape, and tie firmly to the bedstead. Of course the sheet must be placed under the patient in the usual way before inserting the laths. Draw-sheets treated this way will remain smooth much longer than they can do by merely tucking in.

### WORDS OF ADVICE TO NURSES.

"The Hermit Crab" writes: In common with very many of the nursing world, I have read with much interest Miss Young's most able papers on Hints to Hospital Nurses They are very comprehensive, and calculated to bring home to every nurse the sense of her high calling. May I suggest, however, that she leaves unnoticed and unrecognised a vast number of nurses who have joined their profession not wholly out of desire to do good, or for the pure love of mankind, but as an honourable means of earning daily bread? And these, though conscientiously carrying out their duties, and being strictly obedient to their superiors in office (as every gentlewoman would be from an innate sense of self-respect), do not intend to allow their whole lives to become immersed in that "little world" which Miss Young tells us every sister finds in her own ward. I think I speak for very many when I say that it is possible to go through one's daily routine without either unkindness or heartlessness to the patients, and then go off duty and forget that such a place and such people exist. To breathe continuously the atmosphere of hospital life has a particularly narrowing tendency. Sick nurses, in common with the rest of their sex, are prone to discuss each other, their superiors in office, and their medical officers, and I fear that they are no more generous in the way in which they discuss them than are the girls at a large boarding-school when they discuss their mistresses. Therefore, it is as well for our mental health and the freshening of our intellects to dissociate ourselves entirely from the life of the hospital, once off duty, and to become, for the time being, the women we should be had fate blessed us with the good things of this world in sufficient quantities to make work unnecessary. It is wrong to think that one's work suffers—it does not; we merely come back to it with fresh mental stimulus. For this reason I hold that all nursing leagues, nurses' clubs, etc., have a tendency towards cramping and narrowing our lives into a groove from which, if we once allow ourselves to slide into it, it is most difficult to be extricated. This localising tendency is also a great argument against nurses wearing uniform out of doors. When going to or from a case of course it is essential that a nurse should have on her professional garb; but when off duty, if she wear the hospital uniform by compulsion she is not free, and the woman must still come second to the nurse, a trait which should be reserved for the wards alone. A great many nurses in hospitals where outdoor uniforms are optional wear it from pure sloth and disinclination to change into private dress. This, also, is to be deprecated; one's individuality suffers, and it often happens that a girl who enters her training well groomed, and with a knowledge of how to look her best, ends her training a scrupulously clean, but dowdy woman. This letter is very garrulous, but I feel that I must put in an appeal for the very many nurses who do not at all come under the description of an ideal nurse, as given by Miss Young. Do leave us our private lives, and trust us to perform our duties honourably, and be loyal to our authorities, all the more for the fact that they do not constitute our only interests!

### GIFTS.

"An English Nurse" writes: From a nurse's point of view, though not wishing to open a controversy on such a subject as that of gifts to nurses, may I, as one member of that great fraternity, be allowed to object to some of the statements made in The Hospital *Nursing Section* a week or two ago. To begin with. Is it even probable that anyone who, having expressed their appreciation of the nurse who has been with them through a time of trial by some suitable gift, would, when that time lies in the past, turn round and call her a "greedy thing?" Nay, surely, if remembered at all it would more likely be with feelings of kindliness. One of our greatest English writers has said that if we believe human nature to be high and noble, we find it always higher and nobler than we believe it to be, but if we believe it to be low and base we shall find it yet lower and more base. Holding this true I indignantly deny the imputation of coarseness and bad taste without which no nurse could be guilty of boasting of a gift from one patient to another, especially a gift from the rich to one not endowed with many of this world's goods. If the writer of your article has been unfortunate enough to meet such a nurse, would it not have been sufficient to condemn the individual without involving the whole profession in contumely? In such condemnation every right-thinking nurse would most assuredly have joined. Though presents in hospital are generally recognised as impermissible, it frequently happens that the nurse, from no motive of greed, but the higher motive which prompts her to avoid hurting the feelings of the poor and the weak, has to accept gifts of cakes, sticky sweets, etc., to be destroyed in secret afterwards. Who could say that doing thus was a degradation? Rich people likewise have their feelings of gratitude and appreciation, and I cannot see why they should not give, and the nurse receive, without any loss of respect or dignity on either side. Some of the happiest seasons of our lives from childhood upwards are associated with the bestowal of gifts as marks of affection and esteem between relatives and friends. Why not between employer and employed? Would anyone give unless they wanted to give, and would any true nurse allow motives of gain to influence her work? I fear you have formed but a low opinion of nurses, which I sincerely trust that very few people share, for I am convinced that not a small minority but the large majority of nurses are embued with a true enthusiasm for their profession, and perform their duties faithfully and disinterestedly on that account more than for any other reason. I do not believe that the honour of our vocation is becoming shaky in England, but on the contrary, it is being established on a higher basis, for never in the past were the numbers of refined and cultured women to be found in our ranks as at the present day. The questionable allusions to "cabmen" and "touting for tips" are beneath criticism. The cases cannot fairly be considered comparable, for a cabman has not even a bowing acquaintance with his passenger, while a nurse may, and frequently does, become both near and dear to her patient.

### Appointments.

[No charge is made for announcements under this head, and we are always glad to receive, and publish, appointments. But it is essential that in all cases the school of training should be given.]

BOLTON INFIRMARY AND DISPENSARY.—Miss Nancy Jones has been appointed sister-in-charge of Mallett Ward for male surgical patients. She was trained at the Bolton Infirmary.

BRIDGWATER INFIRMARY.—Miss Evelyn Kitching has been appointed matron. She was trained at St. Mary's Hospital, London, and has since been sister of the medical landing at Sir Patrick Dun's Hospital, Dublin, sister of the surgical operation ward and assistant matron at the National Hospital for Paralysis, Queen Square, London. She has also done nursing in South Africa for two years and eight months as a member of the Army Nursing Service Reserve.

BRISTOL ROYAL INFIRMARY.—Miss N. C. Stokes has been appointed night superintendent. She was trained at St. Bartholomew's Hospital, London.

CHESHUNT ISOLATION HOSPITAL.—Miss Gladys Reyner has been appointed matron. She was trained at the City Hospital, Lodge Road, Birmingham, and has since been sister at the Cheshunt Isolation Hospital.

COTTAGE HOSPITAL, AXMINSTER.—Miss Annie Bramwell has been appointed matron. She was trained at Charing Cross Hospital and the City of London Lying-in Hospital, and she has since held the posts of night sister at the Salop Infirmary, night sister at the Children's Infirmary, Liverpool, and matron at the Llandrindod Wells Hospital and Convalescent Home. She holds the L.O.S. certificate.

RICHMOND UNION INFIRMARY.—Miss E. Greene has been appointed assistant nurse. She was trained at Durham County Infirmary, and has since been nurse at the Queen's Jubilee Hospital, Earl's Court, London, and at Northallerton Union Infirmary.

SHREWSBURY INFIRMARY.—Miss Margaret Williams has been appointed night superintendent. She was trained at the Infirmary and Dispensary, Bolton, and upon the completion of her training two years ago was appointed sister-in-charge of the theatre and out-patient department and ward.

SOUTHWARK INFIRMARY, EAST DULWICH GROVE.—Miss Elizabeth Gow Graham has been appointed sister. She was trained at Guy's Hospital, London, and has been nurse at the Hospital for Children, Myrtle Street, Liverpool, and charge nurse at the West Norfolk and Lynn Hospital. She holds the L.O.S. certificate.

### "The Hospital" Convalescent Fund.

THE Hon. Secretary begs to acknowledge, with thanks, the receipt of 2s. 6d. from "E. C. N."

### For Reading to the Sick.

### THE MESSAGE OF SPRING.

SPRING bursts to-day,
For Christ is risen and all the earth's at play.

Flash forth, thou Sun
The rain is over and gone, its work is done.

Winter is past,
Sweet spring is come at last, at last.

Break forth this morn
In roses, thou but yesterday a Thorn.

*C. Rossetti.*

To have faith is to create; to have hope is to call down blessing; to have love is to work miracles. Above all let us see visions, visions of colour and light, of green fields and broad rivers, of palaces laid with fair colours, and gardens where a place is found for rosemary and rue.

While we are still here the language of worship seems far and yet lies very nigh; for what better note can our frail tongues lisp than the voice of wind and sea, river and stream, those grateful servants giving all and asking nothing, the soft whisper of snow and rain eager to replenish, or the thunder proclaiming a majesty too great for utterance? Here, too, stands the angel with the censer gathering up the fragrance of teeming earth and forest-tree, of flower and fruit, and sweetly pungent herb distilled by sun and rain for joyful use. Here, too, come acolytes lighting the dark with tapers —sun, moon, and stars—gifts of the Lord that His sanctuary may stand ever served.

It lies here ready to our hand, this life of adoration which we needs must live hand in hand with earth, for has she not borne the curse with us? But beyond the white gate and the trail of woodbine falls the silence greater than speech, darkness greater than light, a pause of "a little while"; and then the touch of that healing garment as we pass to the King in His beauty, in a land from which there is no return.—*M. Fairless.*

At morn I plucked a rose and gave it Thee
A rose of joy and happy love and peace,
A rose with scarce a thorn:
But in the chillness of a second morn,
My rose bush drooped and all its gay increase
Was but one thorn that wounded me.

I plucked the thorn and offered it to Thee,
And for my thorn Thou gavest love and peace,
Not joy this mortal morn;
If Thou has given such treasure for a thorn
Wilt Thou not give me for my rose increase
Of gladness and all sweets, too?

My thorny rose,

My love and pain

To Thee I offer,

And I set my heart in peace.

*C. Rossetti.*

### Echoes from the Outside World.

### The King's Visit to Malta.

King Edward arrived at Malta after a very enjoyable voyage on Thursday last week. He was conveyed from the Royal yacht to the shore in the State barge amidst the booming of cannon and the clanging of bells, 8,000 school children sang their welcome, and the enthusiasm in Valetta was very great. His Majesty inspected the Guard of Honour in the square opposite the Governor's palace, and in the afternoon held a levée. In the evening, after dining at the palace, he attended the gala performance at the Royal Theatre, the streets *en route* being hung with wreaths of evergreens all lighted by electricity, and lined with troops and great numbers of people. The next day a parade of the troops of the garrison at Marsa took place, though the proceedings were a little spoilt by a sandstorm. The King afterwards visited St. John's Cathedral, where he was received by the Bishop and Chapter, and after luncheon with Admiral Sir C. E. and Lady Domville witnessed a polo match between teams representing the navy and the army. He remained to the end, appearing much interested in the contest. The navy was victorious. The naval review which was to have taken place on Saturday and the water carnival were abandoned because a high wind arose and produced a sandstorm so bad that the function would only have involved great discomfort for all concerned. In the afternoon the King won golden opinions by purposely driving through the poorest parts of the town, so that all his subjects might have an equal chance of seeing him. At night there was a magnificent display of fireworks, and his Majesty, as usual, returned to his yacht to sleep.

Fortunately the weather had completely changed by Monday, the sandstorm having passed, and only soft breezes tempered the rays of the blazing sun. The King landed from his yacht soon after half-past nine, looking much bronzed and in excellent health. The naval force, who were paraded on the Marsa, were 8,000 strong, and the review lasted 50 minutes. Midshipmen on bicycles, clad in white, acted as orderlies, and the *Bacchante's* pet donkey marched in front of the men. In the afternoon the King laid the first stone of the new breakwater, a large concourse of people assembling to witness the ceremony. The postponed water carnival was held in the evening. There were floating models of 13 different types of ships of various periods, ranging from the Ark to the latest battleship, all brilliantly illuminated. They got under weigh soon after 10 o'clock, and included a Chinese war-junk, a Greek galley, a galley of the time of Julius Cæsar, and a model of Columbus's ship the *Pinta*. On the roof of the Ark were Noah and his sons playing stringed instruments, with Noah's wife and daughters in various attire. On passing the Royal vessel the Ark liberated a white dove, which after hovering over the yacht returned to the Ark. As a finale the warships burned red fire and 1,000 rockets were let off. The King left Malta on Tuesday morning with an imposing escort of warships.

### Food Supply in Time of War.

The King has approved the appointment of a Royal Commission to inquire into the conditions affecting the importation of food and raw material into the United Kingdom in time of war, and into the amount of the reserve of such supplies existing in the country at any given period, and to advise whether it is desirable to adopt any measures, in addition to the maintenance of a strong fleet, by which such supplies can be better secured and violent fluctuations of prices avoided. The most notable member is the Prince of Wales, who, in accepting a seat on the Commission, follows the precedent of his father the King, who, as Prince of Wales, was a member of the Royal Commission on the Housing of the Working Classes in 1885.

### The Accident to "Shamrock III."

A disastrous accident happened to *Shamrock III.* on Friday last week, involving the death, by drowning, of one of the yacht's crew. The accident occurred through one of the eyes of the weather rigging screws breaking when the vessel was heeled over by a heavy squall. She was undergoing her sailing trials with *Shamrock I.* off Weymouth when she was dismantled, and at the time of the mishap, which took place about a mile from the pier, both vessels were being watched by hundreds of people who evinced great consternation when they saw the challenger's pyramid of canvas topple bodily over the side of the yacht. When it was ascertained that the life of a steward had been lost and that several persons were also injured, much sympathy and regret were expressed. The owner, Sir Thomas Lipton, was on board. He had just received a pair of binoculars from the steward when he was thrown down the main companion ways, one of the deck hands falling upon the top of him. The binoculars were smashed, but Sir Thomas fortunately escaped with a badly bruised shoulder and a lacerated hand. He afterwards stated that the accident came upon them all without a note of warning, and that he still hopes to fulfil his engagement off Sandy Hook by August 20th. The King from Malta, the Prince of Wales from Sandringham, and others sent telegrams signifying regret at the accident and sympathy with the family of the drowned man.

### Winter in April.

The weather during last week was strangely unseasonable, the temperature one day being registered at 12 degrees colder than the atmosphere on Christmas Day. The frost, in many places severe enough to coat the ponds with thin ice, has naturally done much damage, especially, as owing to the warmth of the previous month, all flowers and fruit trees were very forward. In some parts of the country the blossom of plum, pear, and cherry trees in particular have suffered considerably from the inclemency of the weather, but it is said that the Kentish fruit crop, at any rate in the neighbourhood of Sittingbourne, looks very well, having been hardly hurt at all, because the frost and snow were unaccompanied by cold rain, which does worse damage than even hard frost. Snowstorms have been universal over England and the Continent; at Vienna the suburban tramways had to be suspended because of the accumulated snow; at Berlin the downfall continued for twelve hours, and trees in the gardens of the Royal Palace at Potsdam dating from the time of Frederick the Great were uprooted and hurled to the ground.

### An Invasion of London.

On Saturday the streets of London were invaded by people from the North of England who came to the Capital by excursion trains to see the final of the Association Cup at the Crystal Palace. The specials began to arrive as early as half-past four in the morning at Euston, Marylebone, King's Cross, and St. Pancras; for six hours they kept on arriving, and for the whole of that period a continuous stream poured into the thoroughfares. There were 64,000 persons present on the slopes round the arena at Sydenham, the great proportion of the crowd being heavily rosetted, either with the blue or blue and white of Bury, or the red, and red, white, and black button of Derbyshire. Bands of enthusiasts promenaded wherever there was a clear space, bearing improvised banners with such appeals as "Play up Shakers," "Lancashire's Hope and Pride." Others wore festooned hats or carried parasols of red or blue and white. Many, in defiance of the police, roosted on the tops of trees, and itinerant musicians gained a rich harvest.

### Notes and Queries.

**The Editor is always willing to answer in this column, without any fee, all reasonable questions, as soon as possible.**

**But the following rules must be carefully observed:—**

**1. Every communication must be accompanied by the name and address of the writer.**

**2. The question must always bear upon nursing, directly or indirectly.**

**If an answer is required by letter a fee of half-a-crown must be enclosed with the note containing the inquiry, and we cannot undertake to forward letters addressed to correspondents making inquiries. It is therefore requested that our readers will not enclose either a stamp or a stamped envelope.**

*Indian Army Nursing Service.*

(33) Kindly tell me where I ought to apply for the Indian Nursing Staff?—*Biddy and Nurse W.*

Apply to the India Office, St. James's Park, S.W.

*For the Guidance of Nurses.*

(34) I am desirous of having printed some simple remarks. for the guidance of nurses, for hanging up in enteric wards.—*Medical Superintendent.*

Nurses attending on cases of enteric fever are earnestly requested after every attendance on the patient to be most careful to immediately wash their hands and wrists and rinse them in some disinfectant solution, such as carbolic acid 1–40. Articles, such as feeder, medicine cup, fork, spoon, teacup, etc., used by one patient suffering from enteric fever are on no account to be used by anyone else. Soiled rags, tow, etc., must be burned immediately. Nurses are warned not to indulge in the practice of having biscuits or sweets in their pockets and eating them when in attendance on enteric patients, as in a moment of forgetfulness this might be done while their hands were still not disinfected.

*Asylum.*

(35) Can you kindly tell me of any large asylum near London where a gentleman could be received at moderate charges?—*L. B.*

The Holloway Sanatorium Hospital for the Insane, St. Ann's Heath, Virginia Water, seems suitable.

*Village Nurse.*

(36) I should feel much obliged if you can tell me the address of the Holt-Ockley Nursing Association, and to whom I should apply for preliminary information as to a village nurse.—*Mrs F.*

For information about the Holt-Ockley Association apply to Miss Le Steere, The Cottage, Ockley, Surrey, who will no doubt give you details about village nursing.

*Training.*

(37) Will you kindly tell me if I could get into a London hospital after having been in another hospital, and if 21 is too young to apply?—*E. G.*

Matrons prefer to train their probationers from the beginning, as a rule. Yes, 21 is too young. Learn other things until you are 23

Will you kindly tell me the name of a hospital in Oxford which is a training school for nurses?—*M. B.*

The Radcliffe Infirmary, Oxford, provides a two years' course of training.

*Chapel Rules.*

(38) I am anxious to become a probationer, but I find that at all the hospitals at which I have made inquiries, probationers are required to attend prayers and divine service. Can you tell me of any institution where this is not compulsory?—*M. S.*

This rule is compulsory in nearly all establishments.

*Trilene.*

(39) I should like to know if any reader of The Hospital has tried trilene tablets for stoutness, with what result. I should also like to know, if possible, what they are made of.—*M. C.*

*Truss.*

(40) Would it be possible to obtain a truss for a poor patient from the Surgical Aid Society?—*District Nurse.*

Write and ask the Secretary. The address is Salisbury Square, Fleet Street, E.C.

*Lady Doctor.*

(41) I shall feel obliged if you will oblige me with the name and address of a lady doctor especially skilled in women's diseases. *Miss C.*

We do not give private addresses, but you will find a list of lady doctors attached to the New Hospital for Women, Euston Road, N.W., in "Burdett's Hospitals and Charities."

*Abroad.*

(42) Will you kindly give me the names and addresses of the best hospitals for general training in nursing at Capetown?—*C. V. G.*

The Somerset Hospital, Capetown, is the only general hospital.

*Lepers.*

(43) Will you kindly tell me where there are leper settlements, and if hospital training is necessary in order to obtain a post as attendant?—*K. R.*

You will find a list of leper hospitals in "Burdett's Hospitals and Charities." It is very important that all engaged in attending a contagious disease should be thoroughly versed in modern antiseptic nursing.

*Blind, and Deaf and Dumb.*

(44) Will you kindly tell me where I can obtain particulars for teaching the blind, or the deaf and dumb. Also which are the best homes and schools where one can train for this purpose?—*Adele.*

Possibly the Hon. Secretary, the Royal Normal College for the blind, Weston Street, Upper Norwood, S.E., would give you the desired information as regards the blind, and the Director of the Training College for Teachers of the Deaf, 11 Fitzroy Square, W. that relates to the deaf and dumb.

*Holland Nursing Institute.*

(45) Can you give me the address of the Hollond Nursing Institute?—*A. M.*

The Hollond Institute is now the Nice Nursing Institute, and the address is Villa Pilatte, Avenue Désambrois, Nice.

*Nurses' Clubs.*

(46) Can you give me the name of one or two nurses' clubs in London; and state what are the entrance fees.—*E. P.*

The address of the Trained Nurses' Club is 12 Buckingham Street, Strand, W.C., to which the subscription is 5s. There is also St. Andrew's Club, Mortimer Street, W.

*Lady Roberts' Nursing Service.*

(47) Will you kindly tell me to whom to apply for information respecting Lady Roberts' Nursing Service, and also if it is to be affiliated with Queen Alexandra's Imperial Nursing Service?—*R. H.*

Appointments to the staff of Lady Roberts' nurses are made privately. The service is not incorporated in the Imperial Nursing Service.

*Massage.*

(48) 1. Will you kindly tell me the probable cost, and the time required, to become a fully-trained masseuse? Is it possible to earn one's living in that way without having been trained in any other branch of nursing? Would patients have to be obtained by advertisement or through a doctor? 2. Can you tell me of a home where a harmless mental case could be received for a small payment? The parents have been in better circumstances, but the father is now losing his sight, and the care of this patient is too great a tax upon her sister.—*Leo.*

1. Three months is the average time for the acquisition of the art of massage, and the fees vary from £10 10s. to £40. Work is best obtained through the recommendation of a medical man, and it is quite possible, with a good connection amongst doctors, to earn a living by it. 2. You might write to the Holloway Sanatorium Hospital for the Insane, St. Ann's Heath, Virginia Water, or advertise.

*Free Rooms.*

(49) Can you tell me the best way to obtain a respectable person who, in exchange for two unfurnished rooms in a cottage, in a quiet country place, would look after a relative, aged 64, liable to occasional epileptic fits in her sleep?—*I. G. S.*

We can only advise you to advertise your requirements.

*India.*

(50) Can you give me the name and address of the lady in London who engages nurses for private work in India?—*A. C.*

Possibly you refer to the Up Country Nursing Association. In that case write to Mrs. Sheppard, 10 Chester Place, Regent's Park, N.W.

**Standard Nursing Manuals.**

"The Nursing Profession: How and Where to Train." 2s. net; 2s. 4d. post free.

"Nursing: Its Theory and Practice." (Revised Edition). 3s. 6d. post free.

"Surgical Ward Work and Nursing." (Revised Edition). 3s. 6d. net.; 3s. 10s. post free.

"Practical Handbook of Midwifery." (New Edition). 6s. net; 6s. 3d. post free.

"Notes on Pharmacy and Dispensing for Nurses." 1s. post free.

"Fevers and Infectious Diseases." 1s. post free.

"The Art of Massage." (New Edition). 6s. post free.

### Travel Notes.

**By Our Travelling Correspondent.**

CXX.—ON THE SPANISH FRONTIER.—*Continued from page* 24.

Biarritz is close to St. Jean-de-Luz, only half an hour by train. It is just a gay, uninteresting, and very expensive watering place; a fine sea, but without the picturesque surroundings of St. Jean. One usually conducts one's banking business at Biarritz, and you may like to visit Les Sœurs Silencieuses at Anglet, reached by train. It is an order of more severity even than that of the Trappists for men, and as the nuns are not allowed to exchange a single word with their fellow creatures you are taken round their gloomy dwelling by a sister from a neighbouring convent. How strangely perverted does such a life seem to us—absolutely self-centred—if they do no harm to others they certainly do no good; silent and useless they pass their empty lives in the hope of claiming favour from Heaven.

### Bayonne.

One day you will probably go to Bayonne. The Cathedral is the chief object of attention, but the city is itself of interest from its semi-fortified appearance (you enter through a drawbridge and portcullis), and the memories of the cruel siege in 1813 when both armies suffered so heavily. The streets are narrow and arcaded, and innumerable balconies cling like parasites to the tall, somewhat gloomy houses.

### Walks round St. Jean-de-Luz.

The wild flowers are singularly beautiful in this district in early spring. Violets—gentians (a dwarf sort of extreme brilliancy) darkly blue, contrast vividly with a lovely canary coloured kind of celandine; then on the marshy ground on the banks of the river are found a very lovely kind of narcissus in two shades of yellow, whilst about the end of April gladioli appear in the pools at the foot of St. Barbe. A nice long walk is made by taking train to Biarritz or Guétary, according to pedestrian powers, and returning round the cliffs by the northern side of St. Jean, re-entering the town by St. Barbe.

On the opposite side of the narrow bay is the suburb of Ciboure, the houses all of the same character as St. Jean, and with a church of its own resembling the Basque Cathedral, but not so large or so handsome. Following the beautiful sea road beyond Ciboure you come, after a mile and a half, to the village of Soccoa, with its lighthouse. The coast is very magnificent here and we often brought our tea-basket and picnicked on the cliffs. The elder lady of our party was conveyed, in company with the tea-basket, in a little donkey-cart, drawn by a quadruped of startling activity; on the return journey he invariably bolted, and it was with the utmost difficulty and the united energies of the whole family holding on to his ears and the shafts that our relative was enabled to scramble in; but he was a donkey of placable and engaging disposition and never kicked or deposited us in ditches.

A short distance off (one station only) lies Urrugne—be careful of your pronunciation of this word, or you will be given tickets for Irun, the first Spanish town. It is quite a short walk, about two miles, so you will perhaps walk both ways. Fighting between Soult's and Wellington's troops raged round this village; seven times it was taken and retaken, and in the churchyard the carnage was terrific; now the implacable enemies sleep their last sleep together in that narrow space. You would be interested in reading "The Subaltern" before you go there; he gives a true and most graphic account of the guerilla warfare that took place in and around St. Jean. There is a fine country house called Ithurbide, in Urrugne, and there Marshal Soult had his head-quarters, and at the entrance to St. Jean there is an old convent, now the barracks of the Douaniers, that was used as a hospital for the wounded in that grim time. In the courtyard is an extremely picturesque well and an arcaded cloister surrounds the quadrangle; just outside this are the remains of the original bridge blown up by Marshal Soult; a portion of some of the piers remain. The new bridge is naturally quite uninteresting and much infested by enterprising beggars.

### Sturdy Smugglers at Irun.

You will, no doubt, very frequently cross the frontier into Spain, which by rail is reached through Irun, and really it is a most amusing experience. The Spaniards love to smuggle, very often it seems to me for the mere excitement of the thing. Monsieur l'Abbé had warned us against being made cat's paws at the station, but we had almost forgotten his advice when we met with a practical demonstration of his meaning on our way to St. Sebastian. A fat and comfortable-looking worthy nudged me familiarly in the back as we were going through the turnstile, and on turning round to make inquiries, I was greeted by a knowing wink, whilst at the same moment a silk umbrella (he had two in his hands) was thrust upon me. Almost immediately I grasped the fact that I was being used to smuggle his new umbrella, and promptly returned it to him, turning a deaf and callous ear to his entreaties. On taking our seats (third class) we found ourselves in company with a stout woman carrying a large basket full of drapery which I had watched her pay for, a middle-aged man in peasant blouse, and a mildly innocent boy of 16 or 17. My friend was sitting by the window, and as the train glided out, a man on the platform threw into her lap a bottle of brandy, which was at once grabbed by the mild boy, who volubly explained how his father had passed the customs officers quite boldly with the brandy because he was only going to see him off! No sooner had we fairly started than remarkable transformations took place; the stout woman unblushingly turned up her skirt and denuded herself of three ready-made new petticoats, and the man, rolling up his loose blue trousers, extracted several pieces of thin figured silk such as is used for peasants' handkerchiefs. Over the dividing partition looked a friend who triumphantly produced many yards of gimp trimming. They took us into their confidence, and never seemed to fear for a moment that we should betray them.

---

**Rules in Regard to Correspondence for this Section.**—All questioners must use a pseudonym for publication, but the communication must also bear the writer's own name and address as well, which will be regarded as confidential. All such communications to be addressed "Travel Editor, 'Nursing Section of *The Hospital*,' 28 Southampton Street, Strand." No charge will be made for inserting and answering questions in the inquiry column, and all will be answered in rotation as space permits. If an answer by letter is required, a stamped and addressed envelope must be enclosed, together with 2s. 6d., which fee will be devoted to the objects of "The Hospital" Convalescent Fund. Any inquiries reaching the office after Monday cannot be answered in "The Hospital" of the current week.

## Figures and Tables

**Figure f1:**
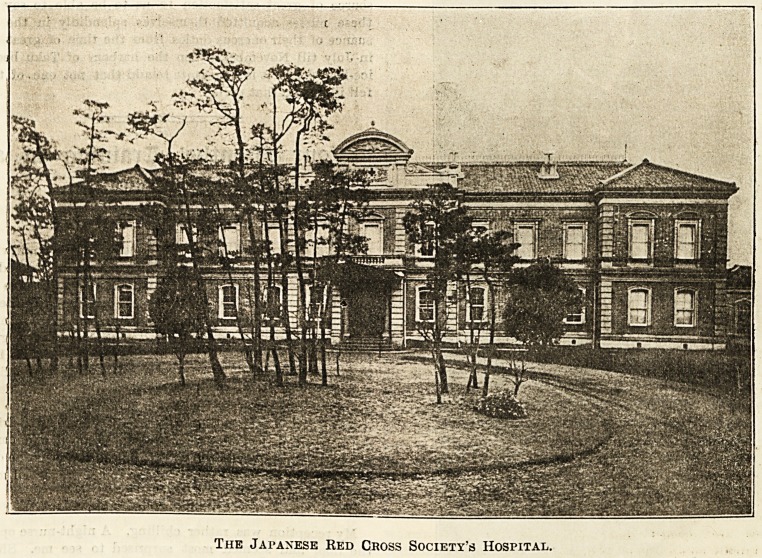


**Figure f2:**
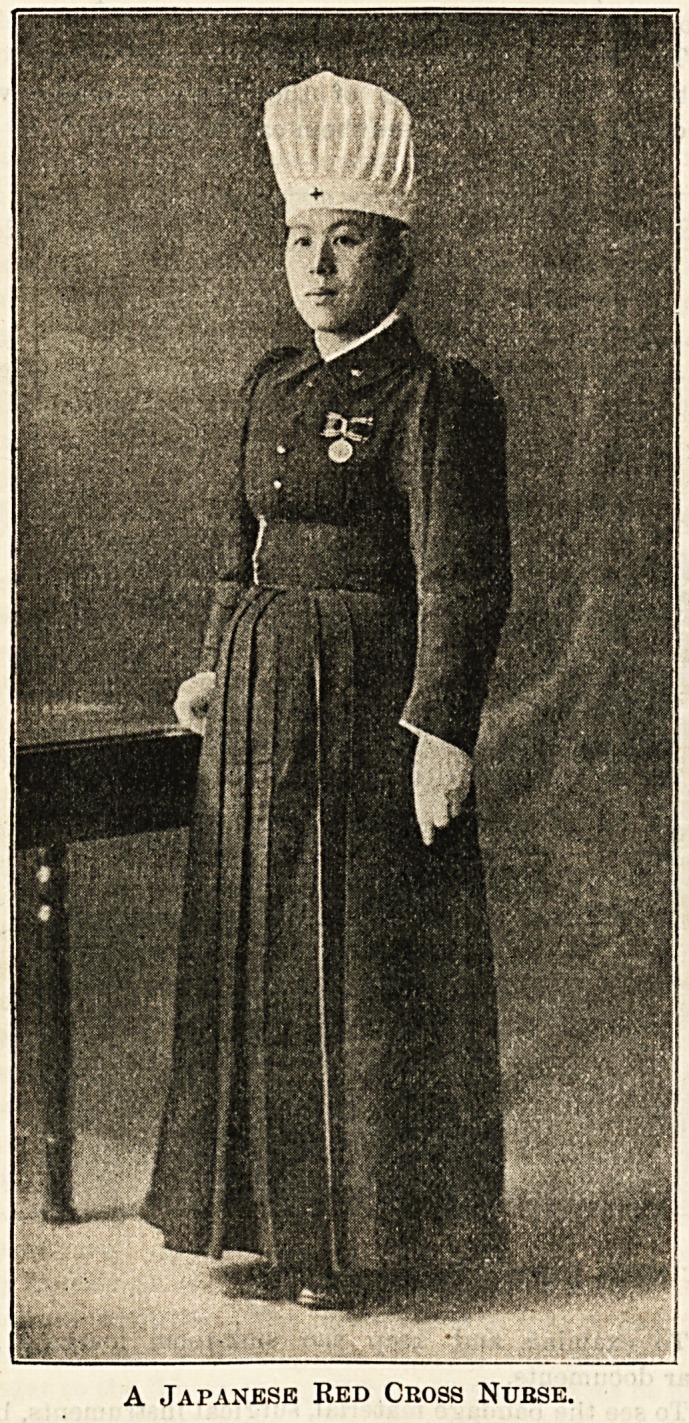


**Figure f3:**